# Structure-Based Bioisosterism Design, Synthesis, Biological Activity and Toxicity of 1,2,4-Oxadiazole Substituted Benzamides Analogues Containing Pyrazole Rings

**DOI:** 10.3390/molecules27154692

**Published:** 2022-07-22

**Authors:** Min-Ting Tu, Ying-Ying Shao, Sen Yang, Bin-Long Sun, Ying-Ying Wang, Cheng-Xia Tan, Xue-Dong Wang

**Affiliations:** 1 College of Chemical Engineering, Zhejiang University of Technology, Hangzhou 310014, China; tt1239803789@163.com (M.-T.T.); s2901692009@163.com (Y.-Y.S.); y17764528124@163.com (S.Y.); zark725@163.com (B.-L.S.); wyy1252909407@163.com (Y.-Y.W.); 2 School of Environmental Science and Engineering, Suzhou University of Science and Technology, Suzhou 215009, China

**Keywords:** 1,2,4-oxadiazole, benzamide compounds, pyrazole, synthesis, biological activity

## Abstract

In order to discover pesticidal lead compounds with high activity and low toxicity, a series of novel benzamides substituted with pyrazole-linked 1,2,4-oxadiazole were designed via bioisosterism. The chemical structures of the target compounds were confirmed via ^1^H NMR, ^13^C NMR and HRMS analysis. The preliminary bioassay showed that most compounds exhibited good lethal activities against *Mythimna separate*, *Helicoverpa armigera*, *Ostrinia nubilalis* and *Spodoptera frugiperda* at 500 mg/L. Particularly in the case of *Mythimna separate*, compound **14q** (70%) exhibited obvious insecticidal activity. In addition, compound **14h** demonstrated good fungicidal activity against *Pyricularia oryae* with an inhibition rate of 77.8%, and compounds **14e**, **14k**, **14n** and **14r** also showed certain antifungal activities (55.6–66.7%). The zebrafish toxicity test showed that the LC_50_ of compound **14h** was 14.01 mg/L, which indicated that it may be used as a potential leading compound for further structural optimization.

## 1. Introduction

**Figure 1:**
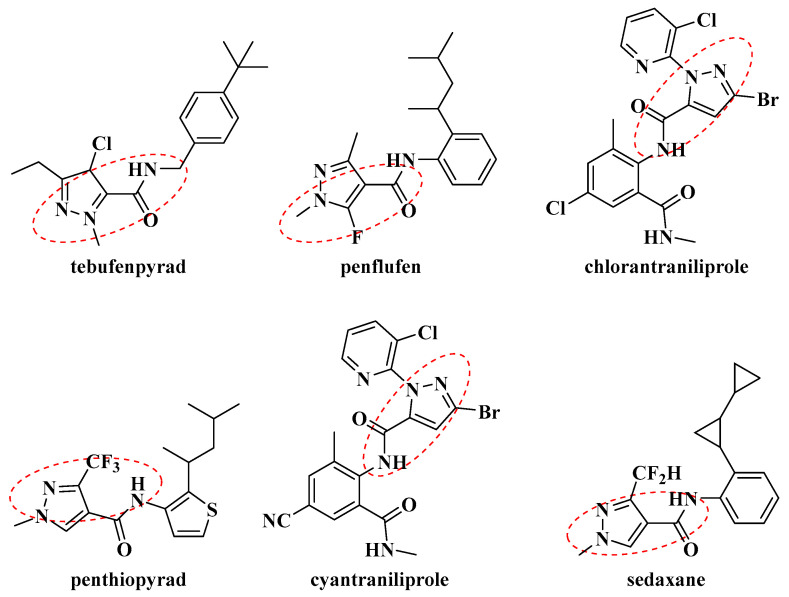
Chemical structures of tebufenpyrad, penflufen, chlorantraniliprole, penthiopyrad, cyantraniliprole and sedaxane.

Nitrogen- and oxygen-containing heterocyclic compounds have become a hotspot in the field of new pesticide development due to their diversity of molecular structures and their breadth of biological activities [1,2,3,4,5,6,7,8]. The 1,2,4-oxadiazole heterocycle, a bioisostere of amides, exhibits certain insecticidal [9,10], antifungal [11,12,13], herbicidal [14], hypotensive [15] and antitumor activities [16] in the biological field. In addition, pyrazoleamides exhibit good insecticidal and fungicidal activities [17,18,19,20], such as tebufenpyrad, penflufen, chlorantraniliprole [21], penthiopyrad [22], cyantraniliprole and sedaxane [23] (Figure 1).

**Figure 2:**
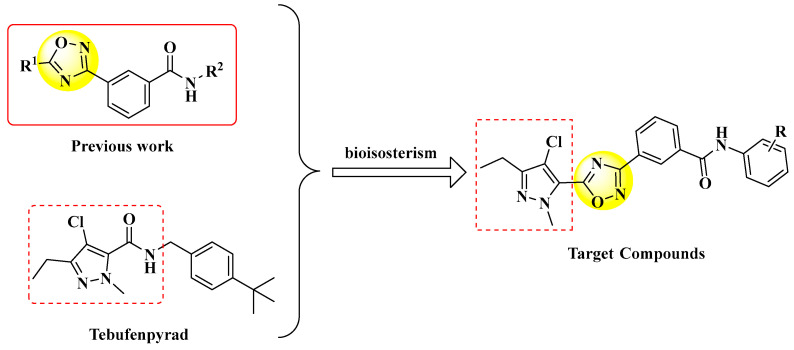
Design strategy for the target compounds.

Our previous studies showed that benzamides substituted with pyridine-linked 1,2,4-oxadiazole derivatives have certain insecticidal and fungicidal activities [24,25]. Therefore, changing amine fragments in pyrazoleamide of tebufenpyrad into 1,2,4-oxadiazole, a series of novel pyrazole-linked 1,2,4-oxadiazoles were designed according to the principle of bioisosterism (Figure 2). The chemical structures of the target compounds were confirmed via ^1^H NMR, ^13^C NMR and HRMS analysis, and their insecticidal activities and fungicidal activities were studied and a toxicity test with zebrafish embryos was performed.

## 2. Results and Discussion

### 2.1. Synthesis of Target Compounds

**Scheme 1:**
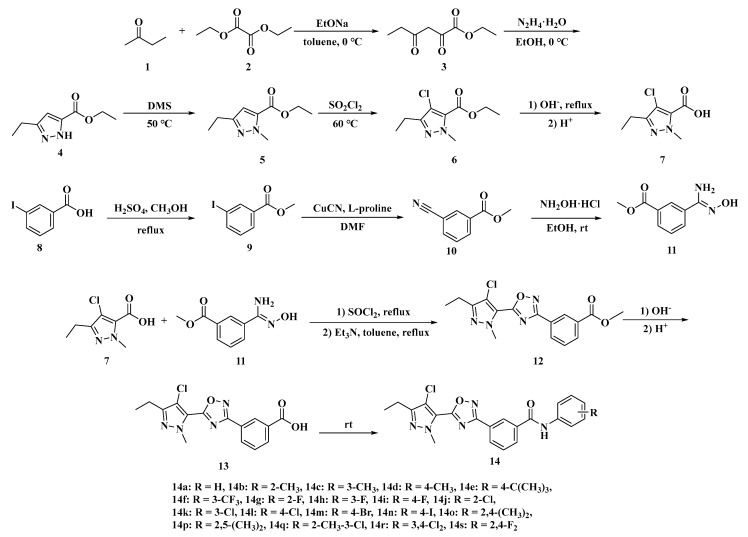
Synthetic route of target compounds **14**.

The synthetic pathways used to target compounds **14a**–**14s** are shown in Scheme 1. The starting material, butanone **1** and diethyl oxalate **2,** experienced Claisen condensation to give ethyl 2,4-dioxohexanoate **3**. Then, this was reacted with N_2_H_4_·H_2_O to give ethyl 3-ethyl-1*H*-pyrazole-5-carboxylate **4**. The reaction of intermediate **4** with dimethyl sulfate (DMS) yielded ethyl 3-ethyl-1-methyl-1*H*-pyrazole-5-carboxylate **5**. Afterwards, compound **5** experienced chlorination and a hydrolysis reaction to give 5-pyrazole acid **7**. Regarding the synthesis of intermediate **11,** refer to our previous work [24]. Finally, the intermediates **7** and **11** went through cyclization, hydrolysis and condensation reactions to obtain the target compounds **14**.

**Scheme 2:**
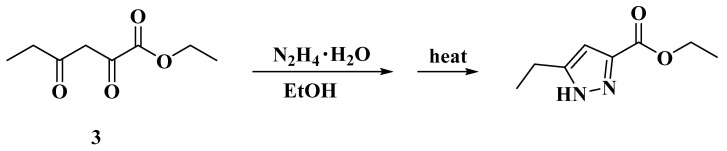
The Knorr cyclization reaction of compound **3**, performed under high temperature.

In step 2, the Knorr cyclization reaction was carried out in an ice bath to avoid the formation of isomers (Scheme 2).

### 2.2. Spectral Analysis of Target Compounds

Compound **14q** was taken as an example to conduct spectral analysis. In the ^1^H NMR spectra of **14q**, the -NH- proton signal was found at δ 10.43 ppm. The -CH- signals of the benzene ring were assigned at δ 8.67–7.29 ppm, and the single peak at δ 4.25 ppm was the peak of N-CH_3_ on the pyrazole ring. The signals at δ 2.65 ppm and δ 1.23 ppm were assigned to -CH_2_ and -CH_3_ of the pyrazole ring, respectively. In addition, the signal of -CH_3_ on the benzene ring was found at δ 2.28 ppm. In the ^13^C NMR spectra of compound **14q**, the appearances of signals at 167.21 ppm and 165.13 ppm were assigned to the carbons of the 1,2,4-oxadiazole ring. In the HRMS spectrogram, the calculated value of the ion peak of this compound was [M + Na]^+^ 456.0989, and the measured value was [M + Na]^+^ 456.0983. The absolute error was within 0.003.

### 2.3. Biological Activities of Target Compounds

The results of the insecticidal activity tests of the target compounds are shown in Table 1. Overall, all the target compounds **14** were found to exhibit certain insecticidal activities against *Mythimna separate*, *Helicoverpa armigera*, *Ostrinia nubilalis* and *Spodoptera frugiperda* at 500 mg/L. Specifically, the mortality rate of compound **14q** against *Mythimna separate* (70%) was higher than the control drug, tebufenpyrad (60%). At the same time, compounds **14a** and **14f** also showed moderate activities (50%). Furthermore, the insecticidal activities of compounds **14** against *Helicoverpa armigera* and *Ostrinia nubilalis* were all below 50%. For *Spodoptera frugiperda*, only compound **14i** exhibited obvious lethality (70%). Moreover, the inhibitory activities of compounds **14** were all below 40% against *Culex pipiens pallens* at 10 mg/L. The structure–activity relationship (SAR) of the target compounds showed that when the substituents of the benzene ring were 4-F and 3-Cl-2-CH_3_, the inhibitory activities against the tested targets were superior to others. Compounds containing F and Cl groups are beneficial to enhance insecticidal activity. Comparing 14b, 14o, 14p and 14q, we can see that Cl was beneficial improving the insecticidal activity of the compound.

The results of the fungicidal activity tests of the target compounds are shown in Table 2. All the target compounds **14** were found to exhibit inhibitory activity against the 10 fungi at 50 mg/L. Compounds **14h** (77.8%), **14e** (55.6%), **14k** (66.7%), **14n** (66.7%) and **14r** (55.6%) showed good inhibitory activities against *Pyricularia oryae*, which were lower than the control drug bixafen (100%). For *Sclerotinia sclerotiorum*, compounds **14g, 14n, 14o, 14p** and **14q** possessed moderately inhibitory activities (45.2%–58.1%). As can be seen, compound **14n** exhibited good inhibitory activity against *Alternaria solani* (50.5%), *Gibberella zeae* (55.9%), *Cercospora arachidicola* (65.9%) and *Riziocotinia solani* (53.3%). From Table 3, we can see that compound **14h** had good inhibitory activity against *Pyricularia oryae* with an EC_50_ of 16.95 mg/L. In addition, by comparing the control effects of compounds **14a**, **14h**, **14k**, **14q** and **14r** on *Pyricularia oryae*, the aniline-containing substituents at the meta position were generally beneficial to improving the fungicidal activity.

### 2.4. Toxicity to Zebrafish Embryo

**Figure 3:**
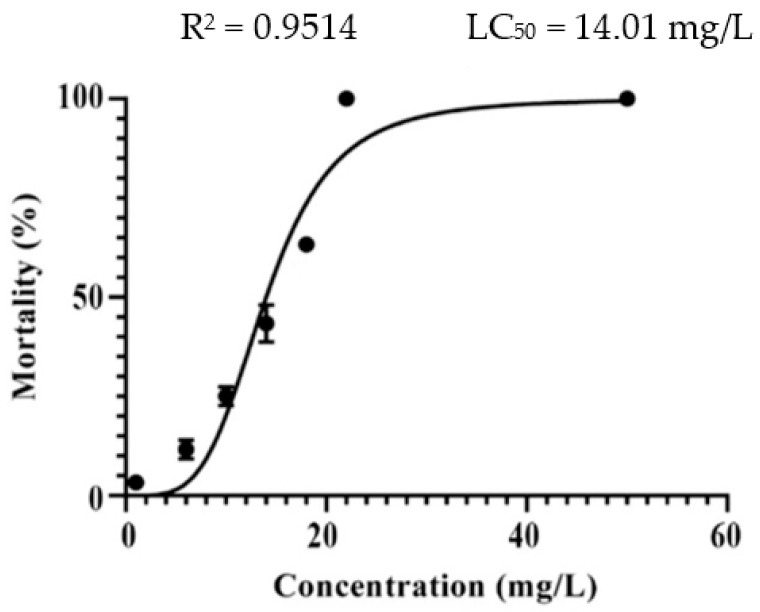
Zebrafish embryo mortality rates after exposure to **14h**.

According to the fungicidal activity results, we selected compound **14h**, which showed good activity, to study the lethal and teratogenic effects of exposure upon zebrafish embryos from 6 to 96 hpf (hours post-fertilization). When the concentration of **14h** was lower than 40 mg/L, the mortality increased sharply with the increase in the concentration. At 40 mg/L, the mortality rate reached as high as 90%. The mortality rate of **14h** showed concentration-dependent curves (Figure 3) with an LC_50_ value of 14.01 mg/L.

**Figure 4:**
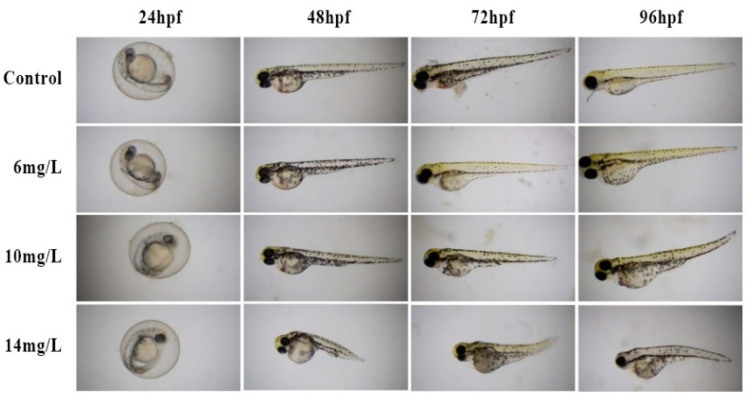
Zebrafish embryo malformation after exposure to compound **14h**.

At 0–24 hpf, zebrafish embryos showed no obvious developmental delay (Figure 4). However, a series of malformations appeared at 48–96 hpf, such as delayed yolk absorption, a significantly shortened body, pericardial cysts, bent spine, melanin deficiency and yolk sac. At 48 hpf, the yolk absorption rate of zebrafish in the 14 mg/L-exposed group was significantly inhibited compared with the control group. At 72 hpf, larval zebrafish exposed at 14 mg/L showed pericardial edema and shortened body lengths. At 96 hpf, bent spines were observed for the larval zebrafish exposed at 10 or 14 mg/L.

## 3. Experimental Section

### 3.1. General Information

Melting points were determined using an X-4 digital microscopic melting point detector (Taike, Beijing, China) and the thermometer was uncorrected. ^1^H NMR and ^13^C NMR spectra were measured on a BRUKER Avance 500 MHz spectrometer (Bruker 500 MHz, Fallanden, Switzerland) using CDCl_3_ or DMSO as the solvent. High-resolution electrospray mass spectra (HR-ESI–MS) were determined using an UPLC H CLASS/QTOF G2 XS mass spectrometer (Waters, Milford, CT, USA). All the reagents were of analytical grade or synthesized in our laboratory. The characterization data for all synthetic compounds are provided in the Supplementary Materials.

Ethics statement: The Institutional Animal Care and Use Committee (IACUC) at Wenzhou Medical University (SYXK 2019-0009, 4 April 2019 to 4 April 2024) approved our study plan for the proper use of zebrafish. All studies were carried out in strict accordance with the guidelines of the IACUC. All dissections were performed on ice, and all efforts were made to minimize suffering.

### 3.2. Synthesis

#### 3.2.1. Ethyl 2,4-Dioxohexanoate **3**

Sodium (2.50 g), toluene (50 mL) and ethanol absolute (30 mL) were added to a three-necked flask successively. Then, the solution of diethyl oxalate (14.63 g, 0.10 mol) in butanone (7.25 g, 0.10 mol) was added dropwise at 0 °C and reacted for 5–6 h. The solvent was removed under reduced pressure and the pH was then adjusted to 2–3 with HCl. Afterwards, the mixture was extracted using toluene and the extraction was dried with MgSO_4_ and filtered. The filtration was concentrated to give 12.70 g yellow liquid. Yield: 73.9%.

#### 3.2.2. Ethyl 3-Ethyl-1*H*-pyrazole-5-carboxylate **4**

N_2_H_4_·H_2_O (4.40 g, 88.50 mmol) was added dropwise to the mixture of ethanol (60 mL) and compounds **3** (12.70 g, 73.80 mmol) at 0 °C to react for 4 h. The solvent was removed under reduced pressure. Then, the residue was extracted using toluene and separated via column chromatography to give 7.20 g light yellow liquid. Yield: 58.2%.

#### 3.2.3. Ethyl 3-Ethyl-1-methyl-1*H*-pyrazole-5-carboxylate **5**

The solution of compound **4** (7.20 g, 0.04 mol) in CHCl_3_ (50 mL) was heated to 35 °C. Then, dimethyl sulfate (7.60 g, 0.06 mol) was added dropwise, and the mixture continued to react at 50 °C for 3 h. At last, purification via column chromatography yielded 6.81 g yellow liquid. Yield: 93.4%. ^1^H NMR (500 MHz, chloroform-d) *δ*: 6.57 (s, 1H), 4.37 (q, *J* = 7.1 Hz, 2H), 3.83 (s, 3H), 2.61 (q, *J* = 8.0 Hz, 2H), 1.37 (t, *J* = 7.1 Hz, 3H), 1.27 (t, *J* = 7.5 Hz, 3H).

#### 3.2.4. Ethyl 4-Chloro-3-ethyl-1-methyl-1*H*-pyrazole-5-carboxylate **6**

The mixture of compound **5** (6.81 g) and CHCl_3_ (50 mL) was heated to 40 °C. Then, SO_2_Cl_2_ (7.60 g, 56.00 mmol) was added dropwise and reacted at 60 °C for 2 h. The mixture was washed with saturated Na_2_CO_3_ and extracted with ethyl acetate and dried with MgSO_4_. Next, the solvent was removed to give 7.53 g solid. The crude product was subjected to the next reaction without further purification.

#### 3.2.5. Intermediate **7**

To a three-necked flask, we added compound **6** (2.10 g 0.01 mol) and ethanol (30 mL), then stirred it to dissolve it completely. Subsequently, NaOH (5 mL, 30%) was added to reflux for 1 h. The solvent was removed and then the pH was adjusted to 2–3 with HCl to precipitate a white solid. The crude product was recrystallized in ethanol and water to afford the pure product.

#### 3.2.6. Intermediate **11**

For the synthesis of intermediate **11**, refer to our previous work [24].

#### 3.2.7. Methyl 3-(5-(4-Chloro-3-ethyl-1-methyl-1*H*-pyrazol-5-yl)-1,2,4-oxadiazol-3-yl)benzoate **12**

The solution of intermediate **7** (0.94 g, 5.00 mmol) and thionyl chloride (10 mL) was added to a three-necked fask and refluxed. Afterwards, the solvent was removed to give 4-chloro-3-ethyl-1-methyl-1*H*-pyrazole-5-carbonyl chloride. Then, the mixture of intermediate **11** (0.97 g, 5.00 mmol), triethylamine (1.20 g, 12.00 mmol) and toluene (100 mL) was stirred at 0 °C for 2 h.

The newly prepared 4-chloro-3-ethyl-1-methyl-1*H*-py-razole-5-carbonyl chloride was added dropwise to react at 0 °C for 3 h, then heated to reflux for 2 h. The mixture was washed with water (150 mL) and a saturated sodium chloride solution, successively. Finally, the organic layer was dried with Na_2_SO_4_ and the solvent was removed to give 1.45 g product. Yield: 57.8%, m.p. 137–139 °C; ^1^H NMR (500 MHz, chloroform-*d*) *δ*: 7.65 (s, 1H), 7.42 (d, *J* = 7.8 Hz, 1H), 7.26 (d, *J* = 7.8 Hz, 1H), 6.86 (t, *J* = 7.8 Hz, 1H), 3.32 (s, 3H), 3.02 (s, 3H), 1.73 (q, *J* = 7.6 Hz, 2H), 0.32 (t, *J* = 7.6 Hz, 3H).

#### 3.2.8. 3-(5-(4-Chloro-3-ethyl-1-methyl-1*H*-pyrazol-5-yl)-1,2,4-oxadiazol-3-yl)benzoic Acid **13**

NaOH (5 mL, 40%) was added to the solution of compound **12** (0.68 g, 2.00 mmol) in THF (40 mL). Then, it was heated to reflux for 2 h and then cooled. THF was removed. Afterwards, 30 mL water was added and the pH was adjusted to 2–3 using HCl to precipitate 0.61 g white solid. Yield: 91.6%, m.p. 183 °C–185 °C.

#### 3.2.9. Target Compounds **14**

Compounds **13** (2.00 mmol) and thionyl chloride (10 mL) were added to a three-necked flask and heated to reflux for 3 h. Then, thionyl chloride was removed under reduced pressure, followed by the addition of THF (30 mL). Afterwards, the solution (2.20 mmol substituted aniline, 5.00 mmol triethylamine, 2 mL THF) was added dropwise at 0–5 °C. This was stirred overnight and purified by means of column chromatography to yield the target compounds **14a**–**14s**.

*3-(5-(4-chloro-3-ethyl-1-methyl-1H-pyrazol-5-yl)-1,2,4-oxadiazol-3-yl)-N-phenylbenzamide,***14a**, white solid, yield 73.3%, m.p. 204 °C–206 °C; ^1^H NMR (500 MHz, DMSO-*d_6_*) *δ* 10.50 (s, 1H), 8.62 (s, 1H), 8.24 (dd, *J* = 31.5, 7.5 Hz, 2H), 7.81 (d, *J* = 7.8 Hz, 2H), 7.77 (t, *J* = 7.7 Hz, 1H), 7.38 (t, *J* = 7.6 Hz, 2H), 7.13 (t, *J* = 7.0 Hz, 1H), 4.24 (s, 3H), 2.63 (q, *J* = 7.4 Hz, 2H), 1.22 (t, *J* = 7.5 Hz, 3H); ^13^C NMR (126 MHz, DMSO-*d_6_*) *δ* 167.67, 167.18, 165.06, 150.38, 139.41, 136.44, 131.32, 130.55, 129.99, 129.09, 126.95, 126.34, 124.87, 124.37, 120.98, 111.71, 41.05, 19.00, 12.93; HRMS calcd. for C_21_H_19_ClN_5_O_2_ [M + H]^+^ 408.1222, found 408.1224.

*3-(5-(4-chloro-3-ethyl-1-methyl-1H-pyrazol-5-yl)-1,2,4-oxadiazol-3-yl)-N-(o-tolyl)benzamide*, **14b**, white solid, yield, 75.3%, m.p. 198 °C–201 °C; ^1^H NMR (500 MHz, DMSO-*d_6_*) *δ* 10.18 (s, 1H), 8.66 (s, 1H), 8.26 (dd, *J* = 25.5, 7.5 Hz, 2H), 7.77 (t, *J* = 7.8 Hz, 1H), 7.37 (d, *J* = 7.7 Hz, 1H), 7.30 (d, *J* = 7.3 Hz, 1H), 7.27–7.18 (m, 2H), 4.24 (s, 3H), 2.63 (q, *J* = 7.6 Hz, 2H), 2.27 (s, 3H), 1.22 (t, *J* = 7.5 Hz, 3H); ^13^C NMR (126 MHz, DMSO-*d_6_*) *δ* 167.71, 167.20, 164.92, 150.39, 136.64, 136.04, 134.27, 131.30, 130.83, 130.57, 130.09, 127.14, 127.00, 126.67, 126.52, 126.39, 124.91, 111.69, 41.05, 19.00, 18.37, 12.96; HRMS calcd. for C_22_H_21_ClN_5_O_2_ [M + H]^+^ 422.1378, found 422.1381.

*3-(5-(4-chloro-3-ethyl-1-methyl-1H-pyrazol-5-yl)-1,2,4-oxadiazol-3-yl)-N-(m-tolyl)benzamide*, **14c**, white solid, yield 75.6%, m.p. 221 °C–224 °C; ^1^H NMR (500 MHz, DMSO-*d_6_*) *δ* 10.42 (s, 1H), 8.61 (s, 1H), 8.23 (dd, *J* = 33.0, 7.8 Hz, 2H), 7.75 (t, *J* = 7.8 Hz, 1H), 7.64 (s, 1H), 7.59 (d, *J* = 8.6 Hz, 1H), 7.25 (t, *J* = 7.8 Hz, 1H), 6.94 (d, *J* = 7.4 Hz, 1H), 4.24 (s, 3H), 2.62 (q, *J* = 7.6 Hz, 2H), 2.32 (s, 3H), 1.21 (t, *J* = 7.6 Hz, 3H); ^13^C NMR (126 MHz, DMSO-*d_6_*) *δ* 167.65, 167.13, 164.96, 150.37, 139.29, 138.26, 136.40, 131.24, 130.49, 129.93, 128.90, 126.92, 126.31, 125.05, 124.82, 121.51, 118.16, 111.72, 41.01, 21.65, 18.99, 12.89; HRMS calcd. for C_22_H_21_ClN_5_O_2_ [M + H]^+^ 422.1378, found 422.1379.

*3-(5-(4-chloro-3-ethyl-1-methyl-1H-pyrazol-5-yl)-1,2,4-oxadiazol-3-yl)-N-(p-tolyl)benzamide*, **14d**, white solid, yield 76.7%, m.p. 215 °C–217 °C; ^1^H NMR (500 MHz, DMSO-*d_6_*) *δ* 10.45 (s, 1H), 8.63 (s, 1H), 8.25 (dd, *J* = 45.5, 7.8 Hz, 2H), 7.78 (t, *J* = 7.8 Hz, 1H), 7.68 (d, *J* = 8.3 Hz, 2H), 7.18 (d, *J* = 8.2 Hz, 2H), 4.26 (s, 3H), 2.66 (q, *J* = 7.5 Hz, 2H), 2.30 (s, 3H), 1.22 (t, *J* = 7.6 Hz, 3H); ^13^C NMR (126 MHz, DMSO-*d_6_*) *δ* 167.73, 167.22, 164.89, 150.40, 136.87, 136.56, 133.38, 131.32, 130.51, 130.04, 129.50, 126.93, 126.35, 124.94, 121.01, 111.69, 41.07, 20.98, 19.01, 12.98; HRMS calcd. for C_22_H_21_ClN_5_O_2_ [M + H]^+^ 422.1378, found 422.1375.

*3-(5-(4-chloro-3-ethyl-1-methyl-1H-pyrazol-5-yl)-1,2,4-oxadiazol-3-yl)-N-(4-(tert-butyl)phenyl)benzamide*, **14e**, white solid, yield 74.8%, m.p. 216 °C–218 °C, ^1^H NMR (500 MHz, DMSO-*d_6_*) *δ* 10.46 (s, 1H), 8.63 (s, 1H), 8.25 (dd, *J* = 41, 7.5 Hz, 2H), 7.78 (t, *J* = 7.7 Hz, 1H), 7.72 (d, *J* = 8.5 Hz, 2H), 7.39 (d, *J* = 8.5 Hz, 2H), 4.26 (s, 3H), 2.65 (q, *J* = 7.5 Hz, 2H), 1.29 (s, 9H), 1.24 (t, *J* = 7.5 Hz, 3H); ^13^C NMR (126 MHz, DMSO-*d_6_*) *δ* 167.72, 167.21, 164.88, 150.40, 146.75, 136.83, 136.53, 131.31, 130.51, 130.01, 126.94, 126.34, 125.71, 124.92, 120.74, 111.70, 41.05, 34.55, 31.67, 19.01, 12.96; HRMS calcd. for C_25_H_2__7_ClN_5_O_2_ [M + H]^+^ 464.1848, found 464.1852.

*3-(5-(4-chloro-3-ethyl-1-methyl-1H-pyrazol-5-yl)-1,2,4-oxadiazol-3-yl)-N-(3-(trifluoromethyl)phenyl)benzamide*, **14f**, yellow solid, yield 65.7%, m.p. 241 °C–243 °C; ^1^H NMR (500 MHz, DMSO-*d_6_*) δ 10.78 (s, 1H), 8.64 (s, 1H), 8.39–8.15 (m, 3H), 8.07 (d, *J* = 8.2 Hz, 1H), 7.78 (t, *J* = 7.0 Hz, 1H), 7.61 (t, *J* = 8.0 Hz, 1H), 7.47 (d, *J* = 7.8 Hz, 1H), 4.24 (s, 3H), 2.63 (q, *J* = 7.5 Hz, 2H), 1.21 (t, *J* = 7.5 Hz, 3H); ^13^C NMR (126 MHz, DMSO-*d_6_*) *δ* 167.60, 167.20, 165.40, 150.37, 140.21, 135.87, 131.37, 130.86, 130.33, 130.02 (d, *J* = 11.5 Hz), 126.97, 126.43, 125.69, 124.85, 124.38, 123.52, 120.63 (d, *J* = 3.7 Hz), 116.99 (d, *J* = 4.5 Hz), 111.72, 41.03, 18.98, 12.90, HRMS calcd. for C_22_H_18_ClF_3_N_5_O_2_ [M + H]^+^ 476.1096, found 476.1093.

*3-(5-(4-chloro-3-ethyl-1-methyl-1H-pyrazol-5-yl)-1,2,4-oxadiazol-3-yl)-N-(2-fluorophenyl)benzamide*, **14g**, yellow solid, yield 63.3%, m.p. 236 °C–238 °C; ^1^H NMR (500 MHz, DMSO-*d_6_*) *δ* 10.43 (s, 1H), 8.66 (s, 1H), 8.27 (dd, *J* = 33.0, 7.8 Hz, 2H), 7.78 (t, *J* = 8.0 Hz, 1H), 7.65 (t, *J* = 8.0 Hz, 1H), 7.33 (d, *J* = 9.5 Hz, 2H), 7.26 (d, *J* = 7.8 Hz, 1H), 4.25 (s, 3H), 2.63 (q, *J* = 7.5 Hz, 2H), 1.22 (t, *J* = 7.5 Hz, 3H); ^13^C NMR (126 MHz, DMSO-*d_6_*) *δ* 167.65, 167.19, 165.05, 157.29, 155.32, 150.38, 135.43, 131.45, 130.83, 130.10, 127.63 (d, *J* = 9.1 Hz), 127.06, 126.40, 125.98 (d, *J* = 12.3 Hz), 124.87, 124.79 (d, *J* = 3.5 Hz), 116.33 (d, *J* = 19.9 Hz), 111.71, 41.03, 18.99, 12.92; HRMS calcd. for C_21_H_18_ClFN_5_O_2_ [M + H]^+^ 426.1128, found 426.1122.

*3-(5-(4-chloro-3-ethyl-1-methyl-1H-pyrazol-5-yl)-1,2,4-oxadiazol-3-yl)-N-(3-fluorophenyl)benzamide*, **14h** yellow solid, yield 61.1%, m.p. 200 °C–203 °C; ^1^H NMR (500 MHz, DMSO-*d*_6_) *δ*: 10.71 (s, 1H), 8.64 (s, 1H), 8.27 (dd, *J* = 32.5, 7.5 Hz, 2H), 7.87–7.74 (m, 2H), 7.59 (d, *J* = 8.2 Hz, 1H), 7.49–7.37 (m, 1H), 6.97 (t, *J* = 10.5 Hz, 1H), 4.27 (s, 3H), 2.66 (q, *J* = 7.5 Hz, 2H), 1.24 (t, *J* = 7.5 Hz, 3H); ^13^C NMR (126 MHz, DMSO-*d*_6_) δ 167.10, 166.68, 164.79, 162.97, 161.05, 149.86, 140.67 (d, *J* = 11.2 Hz), 135.55, 130.85, 130.20 (d, *J* = 10.6 Hz), 129.53, 126.45, 125.89, 124.34, 116.06, 111.22, 110.29 (d, *J* = 20.6 Hz), 107.08 (d, *J* = 26.1 Hz), 40.54, 18.49, 12.41; HRMS calcd. for C_21_H_18_ClFN_5_O_2_ [M + H]^+^ 426.1128, found 426.1127.

*3-(5-(4-chloro-3-ethyl-1-methyl-1H-pyrazol-5-yl)-1,2,4-oxadiazol-3-yl)-N-(4-fluorophenyl)benzamide*, **14i**, yellow solid, yield 66.4%, m.p. 209 °C–213 °C; ^1^H NMR (500 MHz, DMSO-*d_6_*) *δ* 10.56 (s, 1H), 8.62 (s, 1H), 8.28 (d, *J* = 7.7 Hz, 1H), 8.20 (d, *J* = 7.8 Hz, 1H), 7.89–7.68 (m, 3H), 7.22 (t, *J* = 8.7 Hz, 2H), 4.25 (s, 3H), 2.64 (q, *J* = 7.6 Hz, 2H), 1.22 (t, *J* = 7.5 Hz, 3H); ^13^C NMR (126 MHz, DMSO-*d_6_*) *δ* 167.68, 167.21, 165.00, 159.86, 157.95, 150.39, 136.29, 135.74, 131.32, 130.63, 130.06, 126.92, 126.38, 124.90, 122.84 (d, *J* = 7.9 Hz), 115.69 (d, *J* = 22.3 Hz), 111.70, 41.05, 19.00, 12.95; HRMS calcd. for C_21_H_18_ClFN_5_O_2_ [M + H]^+^ 426.1128, found 426.1128.

*3-(5-(4-chloro-3-ethyl-1-methyl-1H-pyrazol-5-yl)-1,2,4-oxadiazol-3-yl)-N-(2-chlorophenyl)benzamide*, **14j**, yellow solid, yield 53.6%, m.p. 198 °C–202 °C; ^1^H NMR (500 MHz, DMSO-*d_6_*) *δ* 10.41 (s, 1H), 8.69 (s, 1H), 8.33 (d, *J* = 8.0 Hz, 1H), 8.25 (d, *J* = 8.0 Hz, 1H), 7.80 (t, *J* = 7.8 Hz, 1H), 7.61 (dd, *J* = 16.0, 7.9 Hz, 2H), 7.43 (t, *J* = 7.7 Hz, 1H), 7.36–7.32 (m, 1H), 4.26 (s, 3H), 2.65 (q, *J* = 7.6 Hz, 2H), 1.23 (t, *J* = 7.5 Hz, 3H), ^13^C NMR (126 MHz, DMSO-*d_6_*) *δ* 167.67, 167.23, 165.08, 150.41, 135.52, 135.35, 131.37, 130.86, 130.20, 130.17, 130.08, 129.09, 128.20, 128.00, 127.05, 126.47, 124.93, 111.70, 41.05, 19.01, 12.96; HRMS calcd. for C_21_H_18_Cl_2_N_5_O_2_ [M + H]^+^ 442.0832, found 442.0836.

*3-(5-(4-chloro-3-ethyl-1-methyl-1H-pyrazol-5-yl)-1,2,4-oxadiazol-3-yl)-N-(3-chlorophenyl)benzamide,***14k***,* white solid, yield 68.3%, m.p. 213 °C–216 °C; ^1^H NMR (500 MHz, DMSO-*d*_6_) *δ*: 10.68 (s, 1H), 8.64 (s, 1H), 8.33 (d, *J* = 7.8 Hz, 1H), 8.22 (d, *J* = 7.8 Hz, 1H), 8.00 (s, 1H), 7.81 (t, *J* = 7.8 Hz, 1H), 7.74 (d, *J* = 9.3 Hz, 1H), 7.42 (t, *J* = 8.1 Hz, 1H), 7.20 (dd, *J* = 7.7, 1.6 Hz, 1H), 4.27 (s, 3H), 2.67 (q, *J* = 7.6 Hz, 2H), 1.24 (t, *J* = 7.6 Hz, 3H); ^13^C NMR (126 MHz, DMSO-*d*_6_) δ 167.61, 167.18, 165.27, 150.37, 140.89, 135.99, 133.43, 131.36, 130.78, 130.05, 126.96, 126.40, 124.85, 124.03, 120.34, 119.22, 111.72, 41.04, 18.99, 12.91; HRMS calcd. for C_21_H_18_Cl_2_N_5_O_2_ [M + H]^+^ 442.0832, found 442.0833.

*3-(5-(4-chloro-3-ethyl-1-methyl-1H-pyrazol-5-yl)-1,2,4-oxadiazol-3-yl)-N-(4-chlorophenyl)benzamide*, **14l**, white solid, yield 74.1%, m.p. 223 °C–225 °C; ^1^H NMR (500 MHz, DMSO-*d_6_*) *δ* 10.65 (s, 1H), 8.63 (s, 1H), 8.31 (d, *J* = 7.5 Hz, 1H), 8.21 (d, *J* = 8.0 Hz, 1H), 7.84 (d, *J* = 8.5 Hz, 2H), 7.79 (t, *J* = 7.7 Hz, 1H), 7.44 (d, *J* = 8.4 Hz, 2H), 4.26 (s, 3H), 2.66 (q, *J* = 7.6 Hz, 2H), 1.23 (t, *J* = 7.4 Hz, 3H); ^13^C NMR (126 MHz, DMSO-*d_6_*) *δ* 167.69, 167.25, 165.21, 150.41, 138.39, 136.23, 131.42, 130.75, 130.12, 129.04, 128.02, 126.96, 126.41, 124.94, 122.49, 111.70, 41.07, 19.01, 12.98; HRMS calcd. for C_21_H_18_Cl_2_N_5_O_2_ [M + H]^+^ 442.0832, found 442.0831.

*3-(5-(4-chloro-3-ethyl-1-methyl-1H-pyrazol-5-yl)-1,2,4-oxadiazol-3-yl)-N-(4-bromophenyl)benzamide*, **14m**, yellow solid, yield 73.3%, m.p. 244 °C–246 °C; ^1^H NMR (500 MHz, DMSO-*d_6_*) *δ* 10.64 (s, 1H), 8.63 (s, 1H), 8.31 (d, *J* = 7.7 Hz, 1H), 8.21 (d, *J* = 7.8 Hz, 1H), 7.88–7.70 (m, 3H), 7.57 (d, *J* = 8.4 Hz, 2H), 4.26 (s, 3H), 2.66 (q, *J* = 7.5 Hz, 2H), 1.23 (t, *J* = 7.4 Hz, 3H); ^13^C NMR (126 MHz, DMSO-*d_6_*) *δ* 167.68, 167.24, 165.22, 150.41, 138.81, 136.22, 131.96, 131.41, 130.76, 130.12, 126.96, 126.41, 124.93, 122.85, 116.11, 111.70, 41.06, 19.01, 12.98; HRMS calcd. for C_21_H_18_BrClN_5_O_2_ [M + H]^+^ 486.0327, found 486.0326.

*3-(5-(4-chloro-3-ethyl-1-methyl-1H-pyrazol-5-yl)-1,2,4-oxadiazol-3-yl)-N-(4-iodophenyl)benzamide*, **14n**, yellow solid, yield 66.3%, m.p. 253 °C–256 °C; ^1^H NMR (500 MHz, DMSO-*d_6_*) *δ* 10.61 (s, 1H), 8.62 (s, 1H), 8.31 (d, *J* = 7.5 Hz, 1H), 8.21 (d, *J* = 7.7 Hz, 1H), 7.79 (t, *J* = 7.7 Hz, 1H), 7.72 (d, *J* = 8.0 Hz, 2H), 7.66 (d, *J* = 8.0 Hz, 2H), 4.26 (s, 3H), 2.66 (q, *J* = 7.7 Hz, 2H), 1.23 (t, *J* = 7.6 Hz, 3H); ^13^C NMR (126 MHz, DMSO-*d_6_*) *δ* 167.68, 167.24, 165.20, 150.41, 139.29, 137.80, 136.25, 131.41, 130.75, 130.11, 126.97, 126.40, 124.93, 123.09, 111.70, 88.14, 41.06, 19.01, 12.98; HRMS calcd. for C_21_H_18_IClN_5_O_2_ [M + H]^+^ 534.0188, found 534.0188.

*3-(5-(4-chloro-3-ethyl-1-methyl-1H-pyrazol-5-yl)-1,2,4-oxadiazol-3-yl)-N-(2,4-dimethylphenyl)benzamide*, **14o**, white solid, yield 68.7%, m.p. 203 °C–205 °C; ^1^H NMR (500 MHz, DMSO-*d_6_*) *δ* 10.10 (s, 1H), 8.66 (s, 1H), 8.29 (d, *J* = 7.5 Hz, 1H), 8.24 (d, *J* = 7.5 Hz, 1H), 7.77 (t, *J* = 8.5 Hz, 1H), 7.23 (d, *J* = 7.5 Hz, 1H), 7.11 (s, 1H), 7.06 (d, *J* = 9.0 Hz 1H), 4.26 (s, 3H), 2.66 (q, *J* = 7.5 Hz, 2H), 2.30 (s, 3H), 2.22 (s, 3H), 1.23 (t, *J* = 8.5 Hz, 3H); ^13^C NMR (126 MHz, DMSO-*d_6_*) *δ* 167.72, 167.17, 164.89, 150.38, 136.10, 135.75, 134.05, 131.34, 131.24, 130.47, 130.02, 127.05, 127.02, 126.98, 126.36, 124.89, 111.69, 41.03, 21.02, 19.00, 18.29, 12.93; HRMS calcd. for C_23_H_23_ClN_5_O_2_ [M + H]^+^ 436.1535, found 436.1533.

*3-(5-(4-chloro-3-ethyl-1-methyl-1H-pyrazol-5-yl)-1,2,4-oxadiazol-3-yl)-N-(2,6-dimethylphenyl)benzamide*, **14p**, white solid, yield 66.6%, m.p. 183 °C–187 °C; ^1^H NMR (500 MHz, DMSO-*d_6_*) *δ* 10.06 (s, 1H), 8.69 (s, 1H), 8.28 (t, *J* = 9.4 Hz, 2H), 7.78 (t, *J* = 7.7 Hz, 1H), 7.15 (s, 3H), 4.25 (s, 3H), 2.63 (q, *J* = 7.6 Hz, 2H), 2.23 (s, 6H), 1.22 (t, *J* = 7.6 Hz, 3H); ^13^C NMR (126 MHz, DMSO-*d_6_*) *δ* 167.70, 167.18, 164.58, 150.38, 136.06, 135.85, 135.54, 131.11, 130.53, 130.11, 128.24, 127.28, 126.88, 126.46, 124.88, 111.69, 41.03, 19.00, 18.53, 12.92; HRMS calcd. for C_23_H_23_ClN_5_O_2_ [M + H]^+^ 436.1535, found 436.1537.

*N-(3-chloro-2-methylphenyl)-3-(5-(4-chloro-3-ethyl-1-methyl-1H-pyrazol-5-yl)-1,2,4-oxadiazol-3-yl)benzamide*, **14q**, yellow solid, yield 62.3%, m.p. 214 °C–216 °C; ^1^H NMR (500 MHz, DMSO-*d_6_*) *δ* 10.43 (s, 1H), 8.67 (s, 1H), 8.31 (d, *J* = 7.8 Hz, 1H), 8.25 (d, *J* = 7.8 Hz, 1H), 7.79 (t, *J* = 7.7 Hz, 1H), 7.38 (dd, *J* = 33.5, 7.8 Hz, 2H), 7.29 (d, *J* = 7.9 Hz, 1H), 4.25 (s, 3H), 2.65 (q, *J* = 7.6 Hz, 2H), 2.28 (s, 3H), 1.23 (t, *J* = 7.5 Hz, 3H); ^13^C NMR (126 MHz, DMSO-*d_6_*) *δ* 167.67, 167.21, 165.13, 150.39, 138.31, 135.64, 134.30, 132.73, 131.37, 130.77, 130.14, 127.42, 127.40, 127.02, 126.43, 126.40, 124.90, 111.70, 41.05, 19.00, 15.83, 12.95; HRMS calcd. for C_22_H_20_Cl_2_N_5_O_2_ [M + H]^+^ 456.0989, found 456.0983.

*3-(5-(4-chloro-3-ethyl-1-methyl-1H-pyrazol-5-yl)-1,2,4-oxadiazol-3-yl)-N-(3,4-dichlorophenyl)benzamide*, **14r**, yellow solid, yield 58.9%, m.p. 194 °C–195 °C; ^1^H NMR (500 MHz, DMSO-*d_6_*) δ 10.74 (s, 1H), 8.62 (s, 1H), 8.31 (d, *J* = 7.7 Hz, 1H), 8.19 (dd, *J* = 15.4, 5.0 Hz, 2H), 7.82–7.71 (m, 2H), 7.63 (d, *J* = 8.8 Hz, 1H), 4.26 (s, 3H), 2.65 (q, *J* = 7.5 Hz, 2H), 1.23 (t, *J* = 7.5 Hz, 3H); ^13^C NMR (126 MHz, DMSO-*d_6_*) *δ* 167.61, 167.23, 165.36, 150.40, 139.54, 135.80, 131.42, 131.36, 131.06, 130.96, 130.16, 126.96, 126.46, 125.85, 124.90, 122.06, 120.85, 111.71, 41.06, 19.00, 12.96; HRMS calcd. for C_21_H_17_Cl_3_N_5_O_2_ [M + H]^+^ 476.0442, found 476.0443.

*3-(5-(4-chloro-3-ethyl-1-methyl-1H-pyrazol-5-yl)-1,2,4-oxadiazol-3-yl)-N-(2,4-difluorophenyl)benzamide*, **14s**, yellow solid, yield 54.7%, m.p. 207 °C–208 °C; ^1^H NMR (500 MHz, DMSO-*d_6_*) *δ* 10.44 (s, 1H), 8.66 (s, 1H), 8.30 (d, *J* = 7.8 Hz, 1H), 8.22 (d, *J* = 7.8 Hz, 1H), 7.78 (t, *J* = 7.8 Hz, 1H), 7.69–7.57 (m, 1H), 7.46–7.31 (m, 1H), 7.16 (t, *J* = 4.3 Hz, 1H), 4.25 (s, 3H), 2.63 (q, *J* = 7.5 Hz, 2H), 1.22 (t, *J* = 7.5 Hz, 3H); ^13^C NMR (126 MHz, DMSO-*d_6_*) *δ* 167.63, 167.20, 165.12, 161.26 (d, *J* = 11.6 Hz), 159.31 (d, *J* = 11.2 Hz), 157.58 (d, *J* = 12.4 Hz), 155.59 (d, *J* = 12.6 Hz), 150.38, 135.21, 131.16 (d, *J* = 67.0 Hz), 130.14, 129.02 (d, *J* = 9.9 Hz), 127.02, 126.43, 124.87, 122.53 (dd, *J* = 12.4, 3.5 Hz), 111.70, 104.89 (t, *J* = 25.2 Hz), 41.03, 18.99, 12.92; HRMS calcd. for C_21_H_17_ClF_2_N_5_O_2_ [M + H]^+^ 444.1033, found 444.1035.

## 4. Conclusions

In conclusion, a series of novel pyrazole-linked 1,2,4-oxadiazoles were designed by means of bioisosterism. The preliminary bioassay showed that most compounds exhibited good lethal activities against *Mythimna separate*, *Helicoverpa armigera*, *Ostrinia nubilalis* and *Spodoptera frugiperda* at 500 mg/L. Specifically, for *Mythimna separate*, compound **14q** (70%) exhibited obvious insecticidal activity. At 50 mg/L, compound **14h** (77.8%) displayed fungicidal activity against *Pyricularia oryae*. In addition, the acute toxicity of **14h** to zebrafish embryos was 14.01 mg/L, and it was thus classified as a low-toxicity compound. Therefore, these compounds could potentially be selected as lead compounds for further studies.

## Figures and Tables

**Table 1:** Insecticidal activities of compounds **14a**–**14s**.

Compounds	R	Insecticidal Activities (Death Rates %)				
		*Mythimna separate* 500 mg/L	*Helicoverpa armigera* 500 mg/L	*Ostrinia nubilalis* 500 mg/L	*Spodoptera frugiperda* 500 mg/L	*Culex pipiens pallens* 10 mg/L
**14a**	H	50	20	10	0	10
**14b**	2-CH_3_	20	10	5	10	0
**14c**	3-CH_3_	30	10	10	5	25
**14d**	4-CH_3_	10	15	30	0	0
**14e**	4-t-Bu	20	25	40	0	5
**14f**	3-CF_3_	50	35	50	15	20
**14g**	2-F	25	30	45	0	5
**14h**	3-F	30	35	50	20	0
**14i**	4-F	35	20	30	70	30
**14j**	2-Cl	15	25	40	25	0
**14k**	3-Cl	40	10	0	10	15
**14l**	4-Cl	30	15	20	30	5
**14m**	4-Br	5	0	0	0	0
**14n**	4-I	20	10	5	0	10
**14o**	2,4-di-CH_3_	10	15	15	20	25
**14p**	2,6-di-CH_3_	20	15	10	30	0
**14q**	3-Cl-2-CH_3_	70	45	15	40	25
**14r**	3,4-di-Cl	40	10	5	10	0
**14s**	2,4-di-F	50	20	10	0	15
Tebufenpyrad		60	45	40	30	45

Note: All the data were determined three times.

**Table 2:** Fungicidal activities of compounds **14a**–**14s** at 50 mg/L.

Compounds	R	Fungicidal Activities (Inhibition Rate %)									
		*AS*	*GZ*	*PO*	*PC*	*SS*	*BC*	*RS*	*FO*	*CA*	*PP*
**14a**	H	21.4	17.6	33.3	18.8	16.1	11.4	24.4	17.4	13.3	19.4
**14b**	2-CH_3_	21.4	26.5	22.2	9.4	29.0	13.6	22.0	8.7	6.7	8.3
**14c**	3-CH_3_	21.4	32.4	55.6	18.8	32.3	18.2	4.9	8.7	20.0	25.0
**14d**	4-CH_3_	28.6	44.1	44.4	18.8	9.7	4.5	12.2	17.4	13.3	8.3
**14e**	4-t-Bu	21.4	38.2	55.6	12.5	16.1	18.2	4.9	8.7	6.7	8.3
**14f**	3-CF_3_	21.4	23.5	11.1	12.5	29.0	22.7	7.3	8.7	6.7	11.1
**14g**	2-F	21.4	23.5	33.3	9.4	45.2	13.6	22.0	8.7	40.0	11.1
**14h**	3-F	21.4	35.3	77.8	3.1	38.7	13.6	4.9	8.7	6.7	2.8
**14i**	4-F	7.1	44.1	33.3	3.1	32.3	18.2	12.2	8.7	26.7	30.6
**14j**	2-Cl	21.4	14.7	44.4	3.1	41.9	22.7	36.6	4.3	40.0	33.3
**14k**	3-Cl	14.3	35.3	66.7	12.5	38.7	13.6	4.9	4.3	6.7	13.9
**14l**	4-Cl	14.3	32.4	11.1	12.5	32.3	18.2	36.6	4.3	33.3	11.1
**14m**	4-Br	7.1	17.6	11.1	12.5	38.7	13.6	12.2	4.3	26.7	30.6
**14n**	4-I	50.0	55.9	66.7	12.5	58.1	31.8	65.9	13.0	53.3	30.6
**14o**	2,4-di-CH_3_	21.4	35.3	11.1	12.5	58.1	31.8	48.8	8.7	60.6	25.0
**14p**	2,6-di-CH_3_	28.6	38.2	33.3	9.4	51.6	27.3	4.9	8.7	46.7	22.2
**14q**	3-Cl-2-CH_3_	21.4	32.4	44.4	18.8	48.4	40.9	31.7	4.3	40.0	22.2
**14r**	3,4-di-Cl	21.4	17.6	55.6	9.4	25.8	31.8	24.4	8.7	6.7	11.1
**14s**	2,4-di-F	21.4	23.5	44.4	3.1	32.3	22.7	46.3	8.7	40.0	11.1
Bixafen		92.9	70.6	100.0	40.6	100.0	72.7	92.7	73.9	86.7	77.8

Note: *Alternaria solani* (*AS*), *Gibberella zeae* (*GZ*), *Pyricularia oryae* (*PO*), *Phytophthora capsica* (*PC*), *Sclerotinia sclerotiorum* (*SS*), *Botrytis cinerea* (*BC*), *Riziocotinia solani* (*RS*), *Fusarium oxysporum* (*FO*), *Cercospora arachidicola* (*CA*), *Physalospora piricola* (*PP*). All the data were determined three times.

**Table 3:** EC_50_ of compound **14h** and bixafen to *Pyricularia oryae* (*PO*).

Compounds	y = a + bx	r^2^	EC_50_/(mg·L^−1^)
**14h**	y = 1.6022x + 3.0305	0.9968	16.95
bixafen	y = 1.7973x + 3.2716	0.9766	9.15

## Data Availability

The data presented in this study are available in the article and Supplementary Materials.

## Supplementary Materials

The following supporting information can be downloaded at: https://www.mdpi.com/article/10.3390/molecules27154692/s1, Figures S1–S19: ^1^H NMR spectra of **14a**–**14s**; Figures S20–S38: ^13^C NMR spectra of **14a**–**14s**; Figures S39–S57: ESI-HRMS spectra of **14a**–**14s**.

## Sample Availability

Samples of the compounds are not available from the authors.
