# Supplementary material for: Structure-Based Bioisosterism Design, Synthesis, Biological Activity and Toxicity of 1,2,4-Oxadiazole Substituted Benzamides Analogues Containing Pyrazole Rings
Source: Molecules. 2022 Jul 22;27(15):4692. doi: 10.3390/molecules27154692 (PMC9330712; doi:10.3390/molecules27154692)
Supplement: Supplementary file 1 [file molecules-27-04692-s001.zip › molecules-1818533-supplementary.pdf]

Supplementary Materials

Structure-Based Bioisosterism Design, Synthesis, Biological Activity and Toxicity of 1,2,4-Oxadiazole Substituted Benzamides Analogues Containing Pyrazole Rings

Min-Ting Tu, Ying-Ying Shao, Sen Yang, Bin-Long Sun, Ying-Ying Wang, Cheng-Xia Tan * and Xue-Dong Wang*

1. ^{1}H NMR spectra of **14a~14s**……………………………………S1-S19
2. ^{13}C NMR spectra of **14a~14s**…………………………………S20-S38
3. ESI-HRMS spectra of **14a~14s**………………………………S39-S57

1. ^{1}H NMR spectra of **14a~14s**

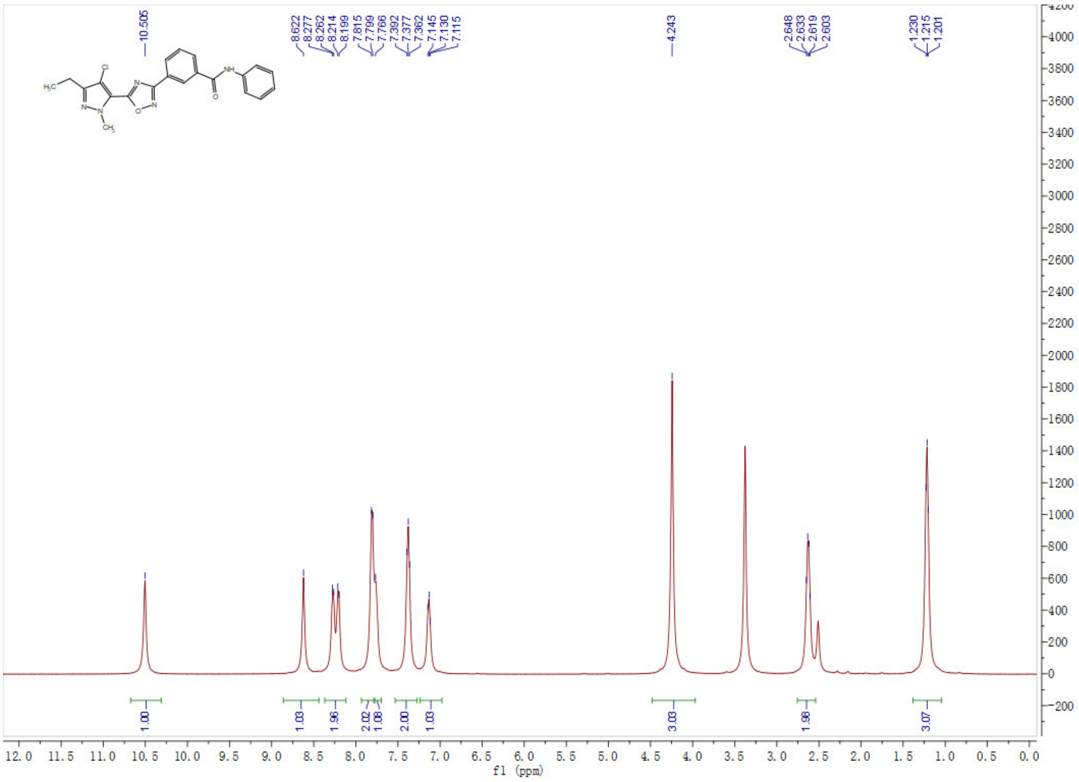

Figure S1. ^{1}H NMR spectra of **14a**

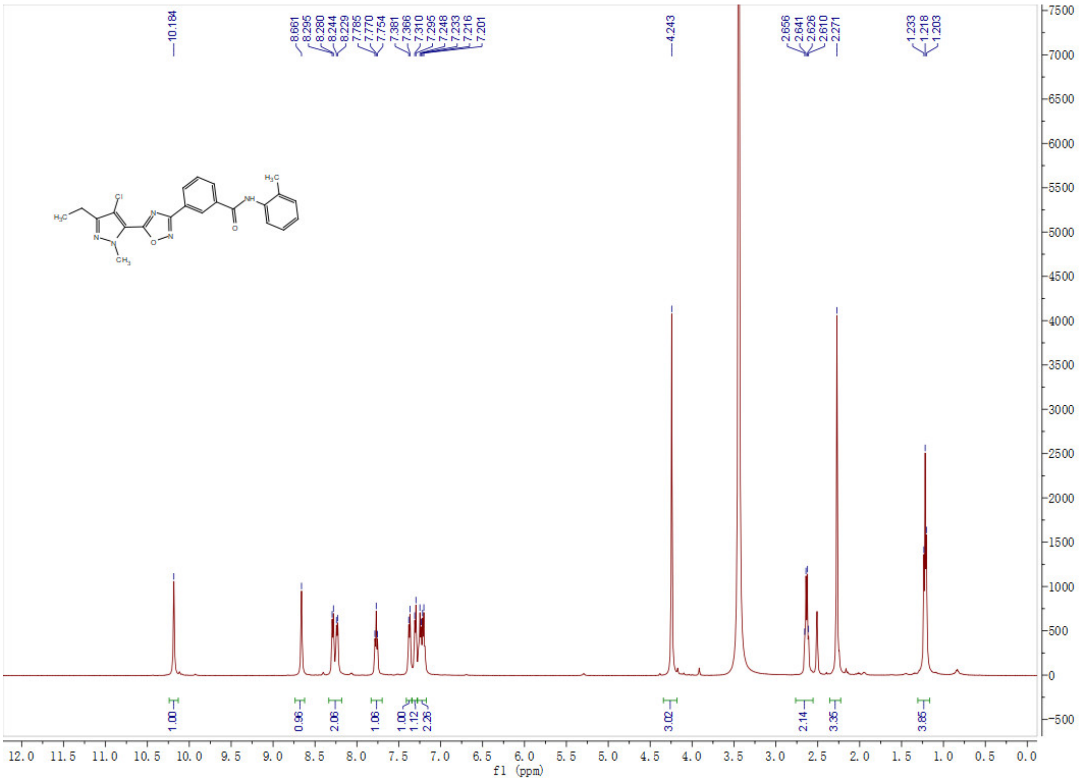

Figure S2. ^{1}H NMR spectra of **14b**

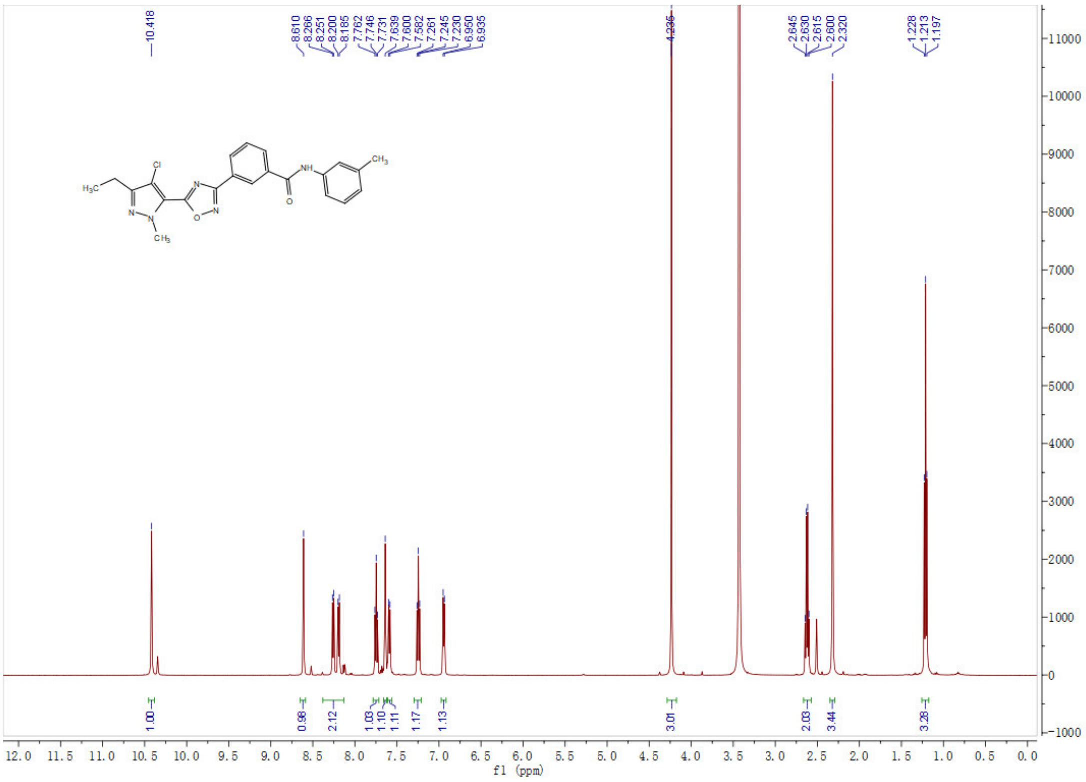

Figure S3. ^{1}H NMR spectra of **14c**

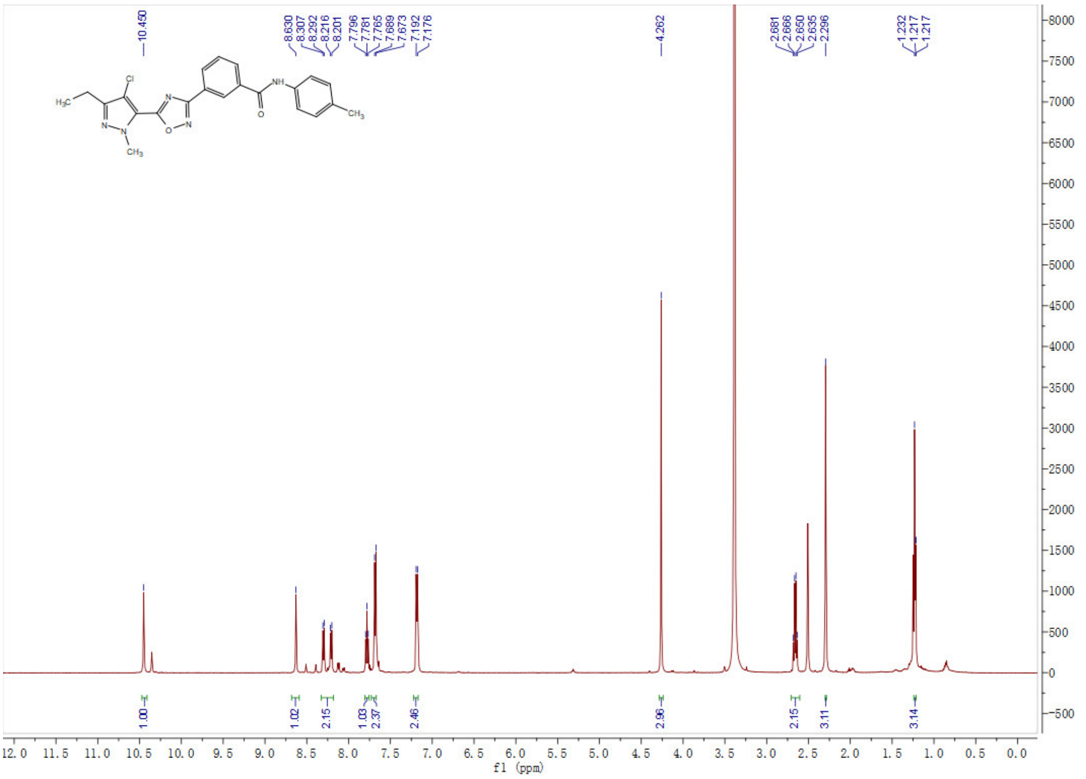

Figure S4. ^{1}H NMR spectra of **14d**

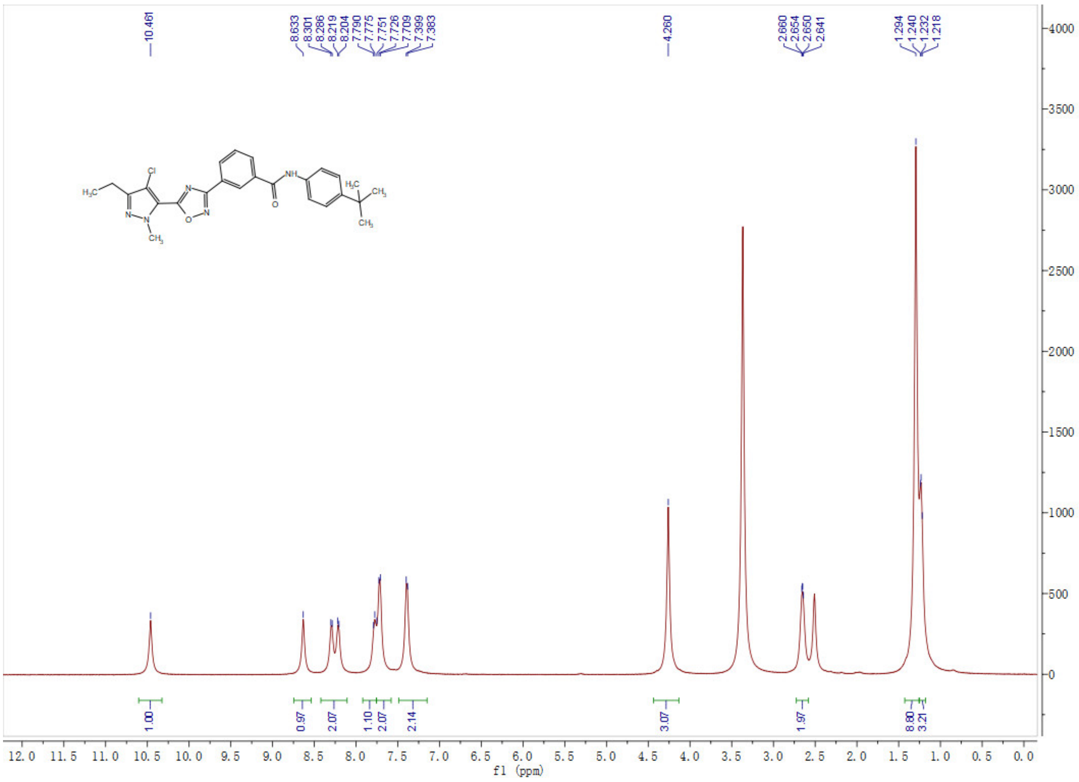

Figure S5. ^{1}H NMR spectra of **14e**

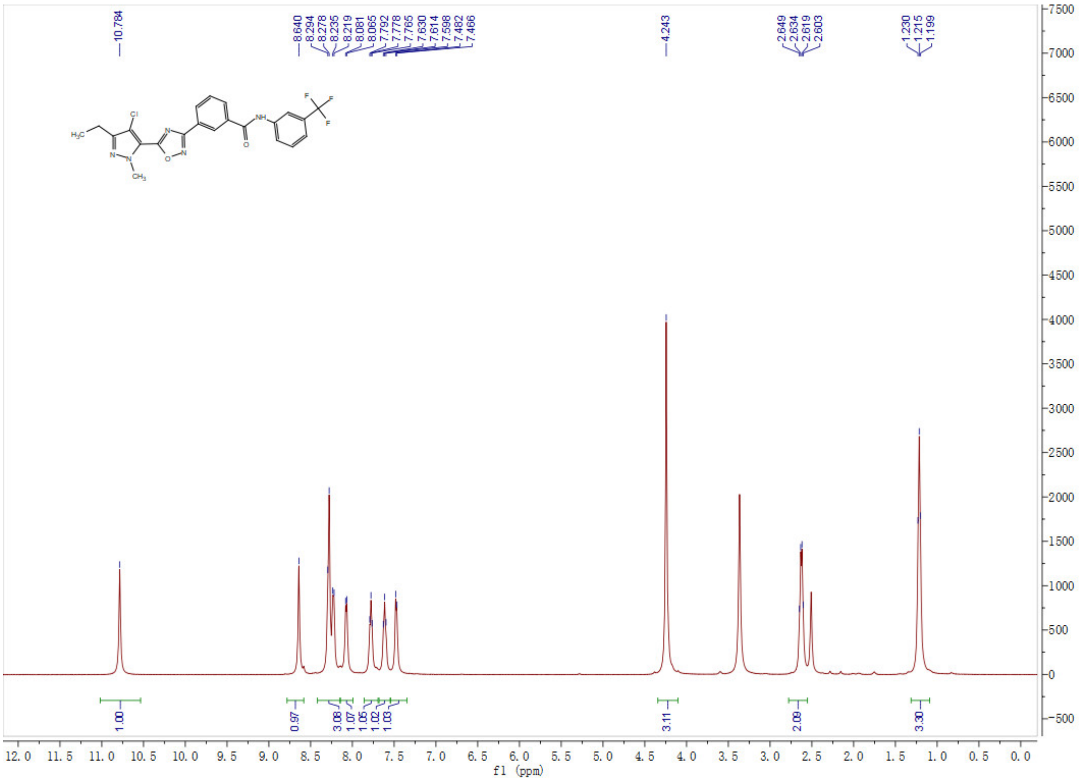

Figure S6. ^{1}H NMR spectra of **14f**

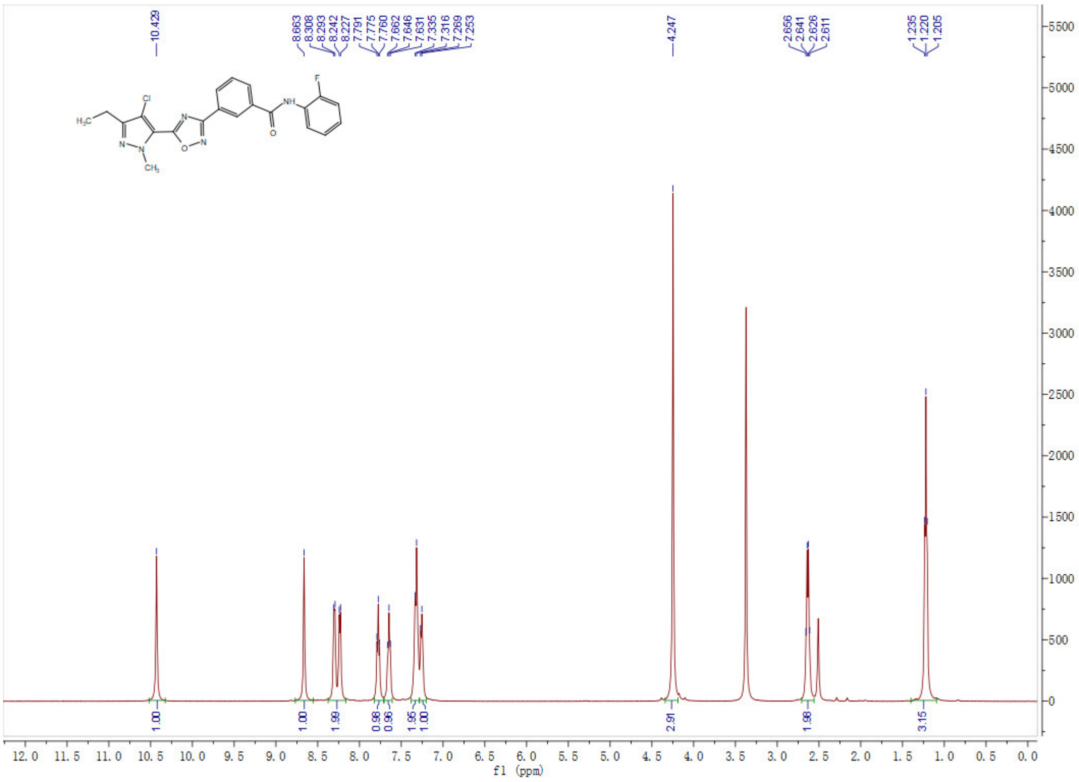

Figure S7. ^{1}H NMR spectra of **14g**

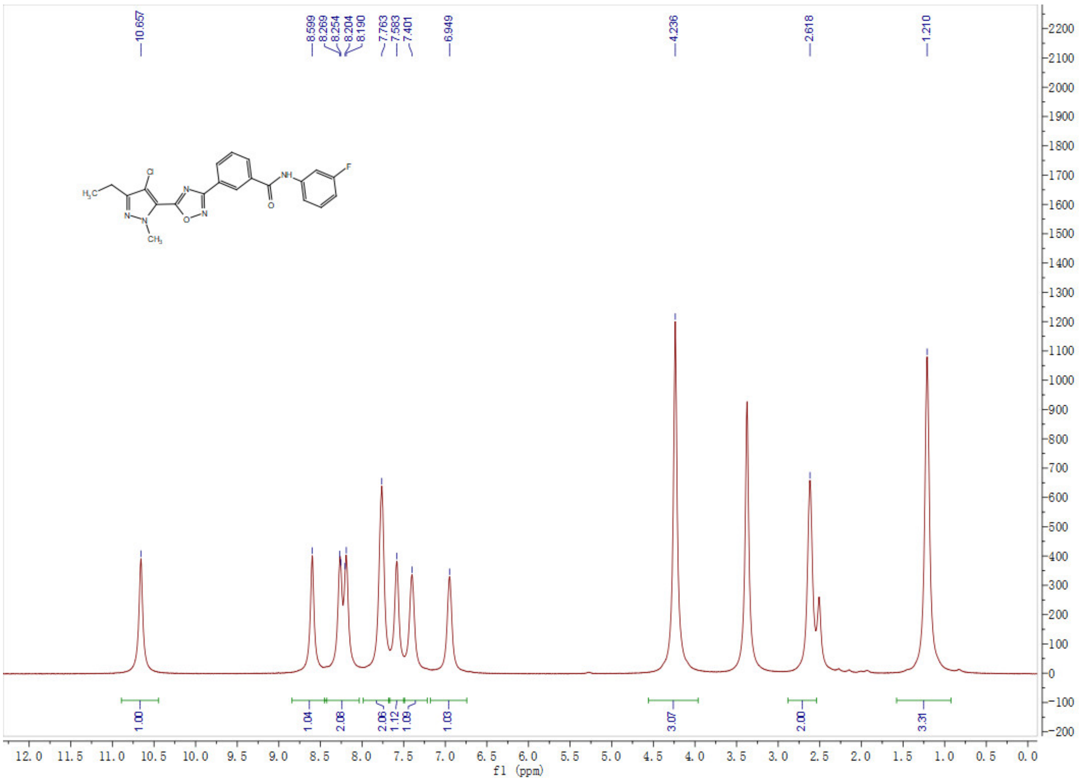

Figure S8. ^{1}H NMR spectra of **14h**

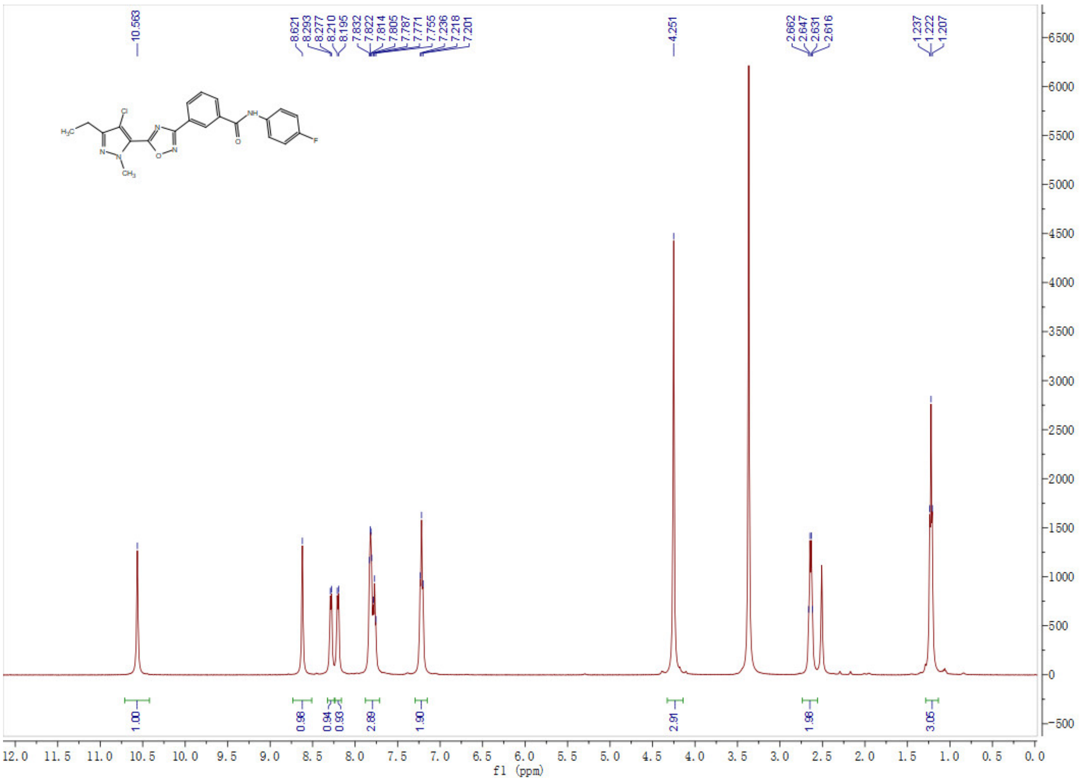

Figure S9. ^{1}H NMR spectra of **14i**

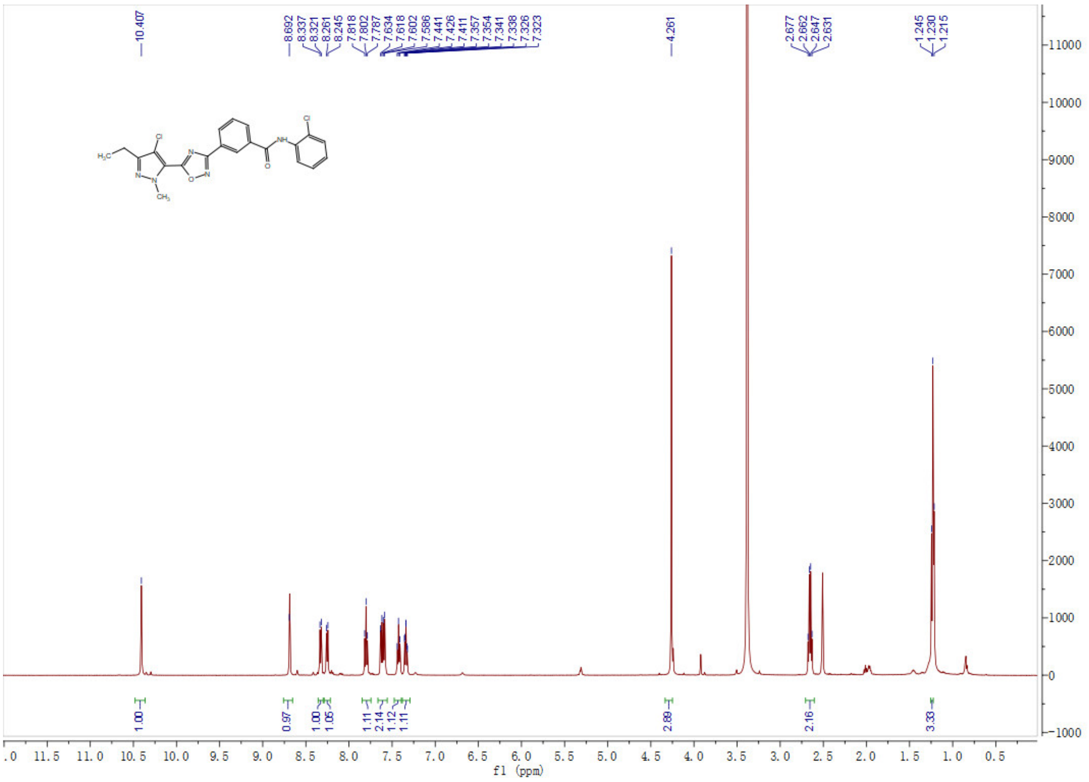

Figure S10. ^{1}H NMR spectra of **14j**

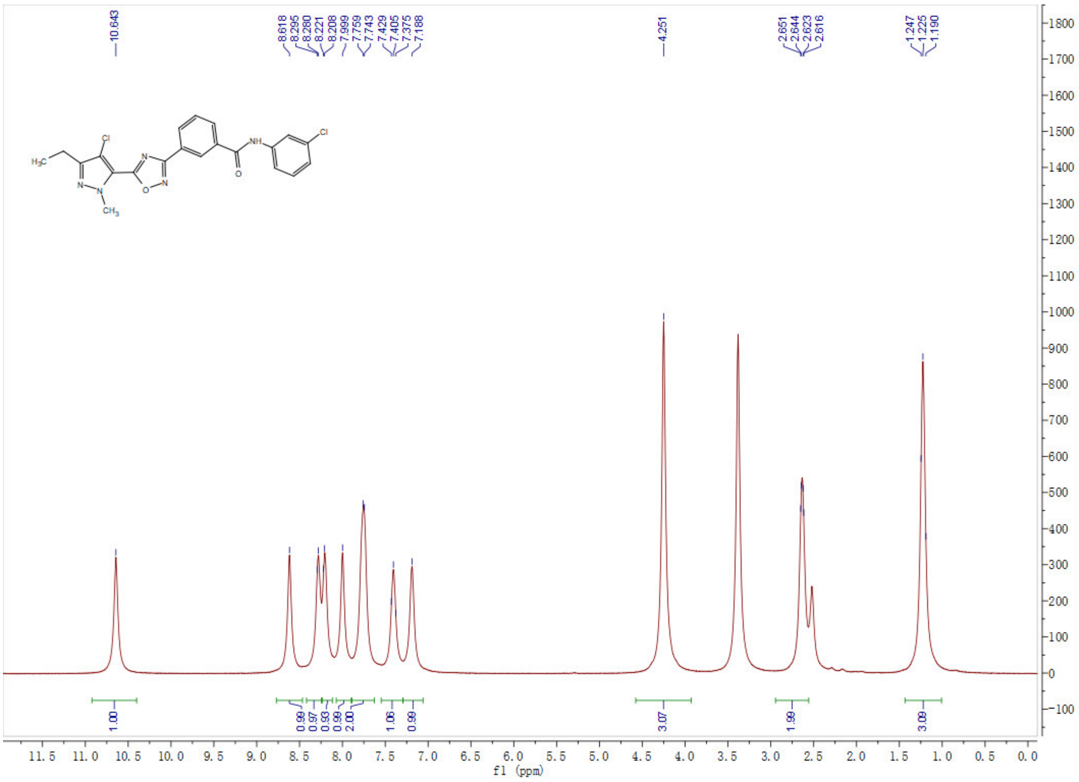

Figure S11. ^{1}H NMR spectra of **14k**

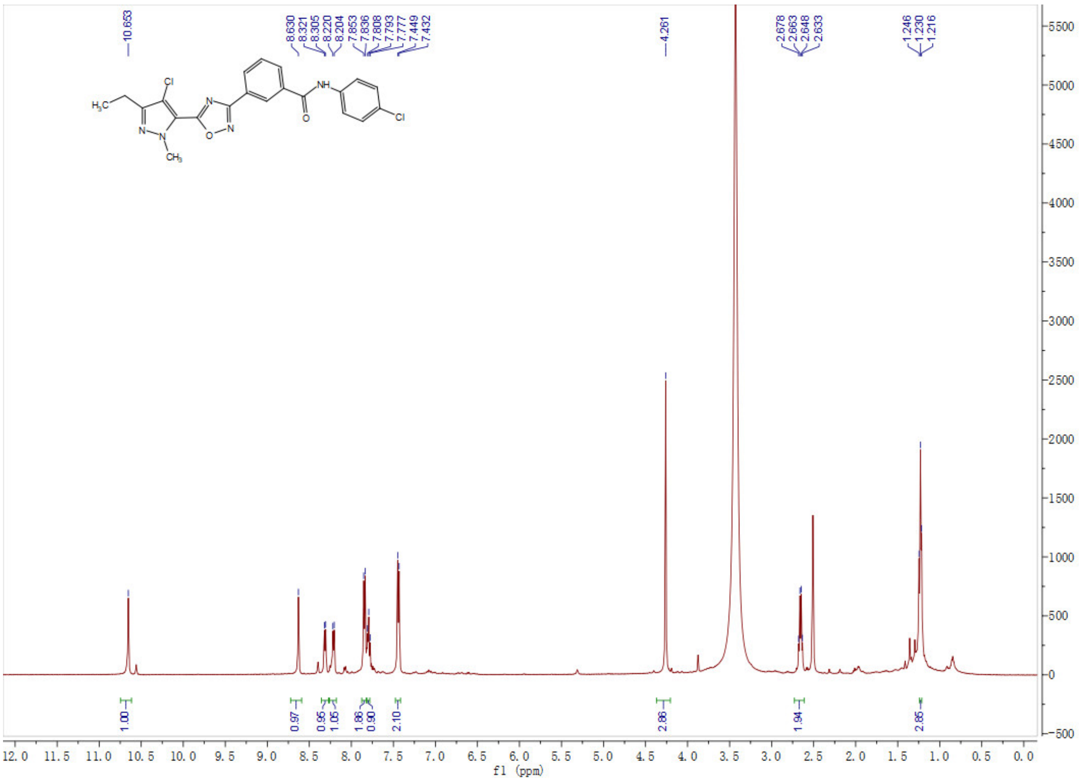

Figure S12. ^{1}H NMR spectra of **14l**

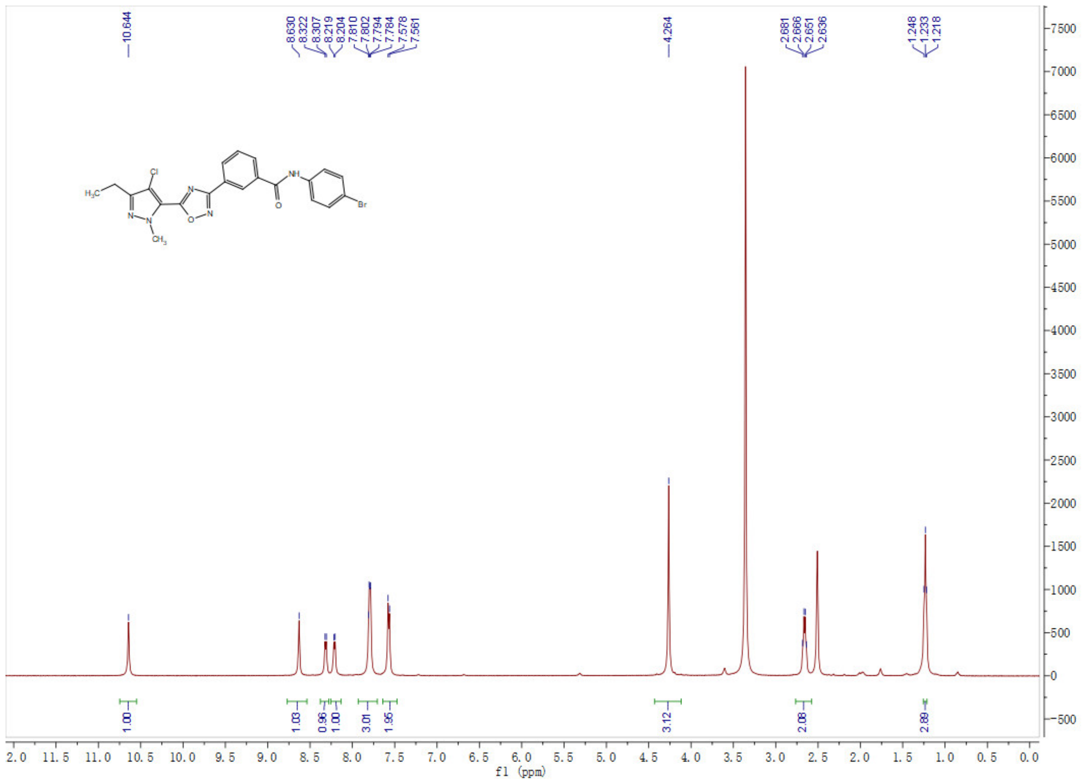

Figure S13. ^{1}H NMR spectra of **14m**

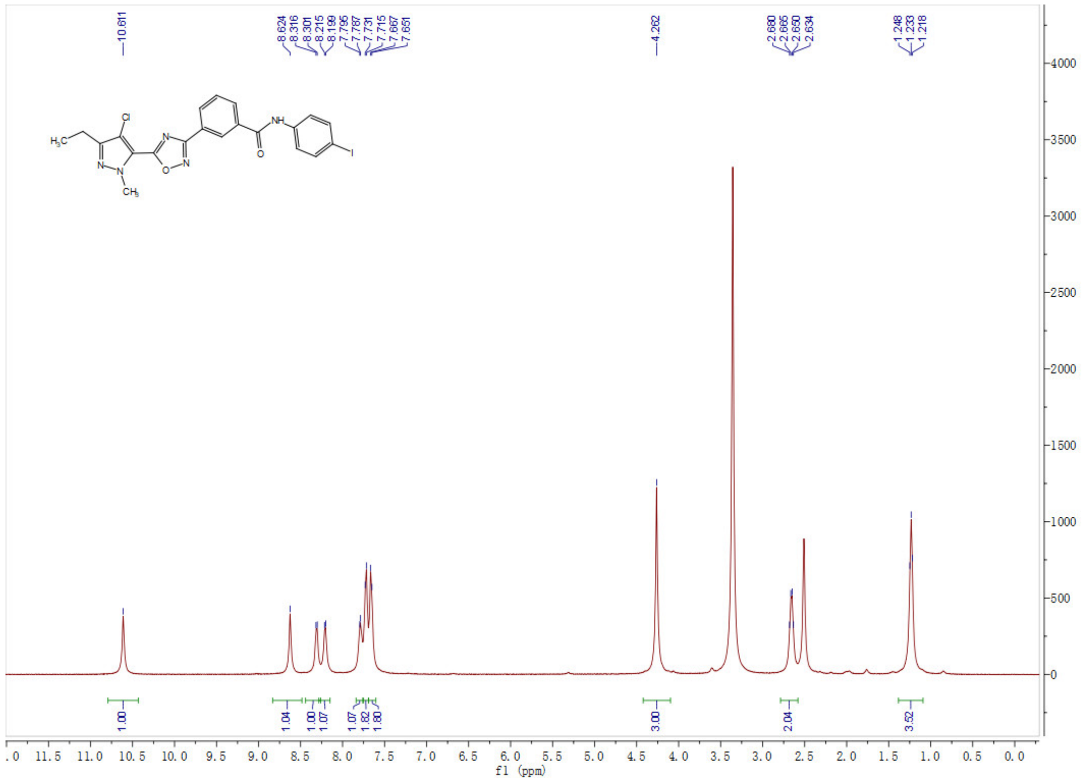

Figure S14. ^{1}H NMR spectra of **14n**

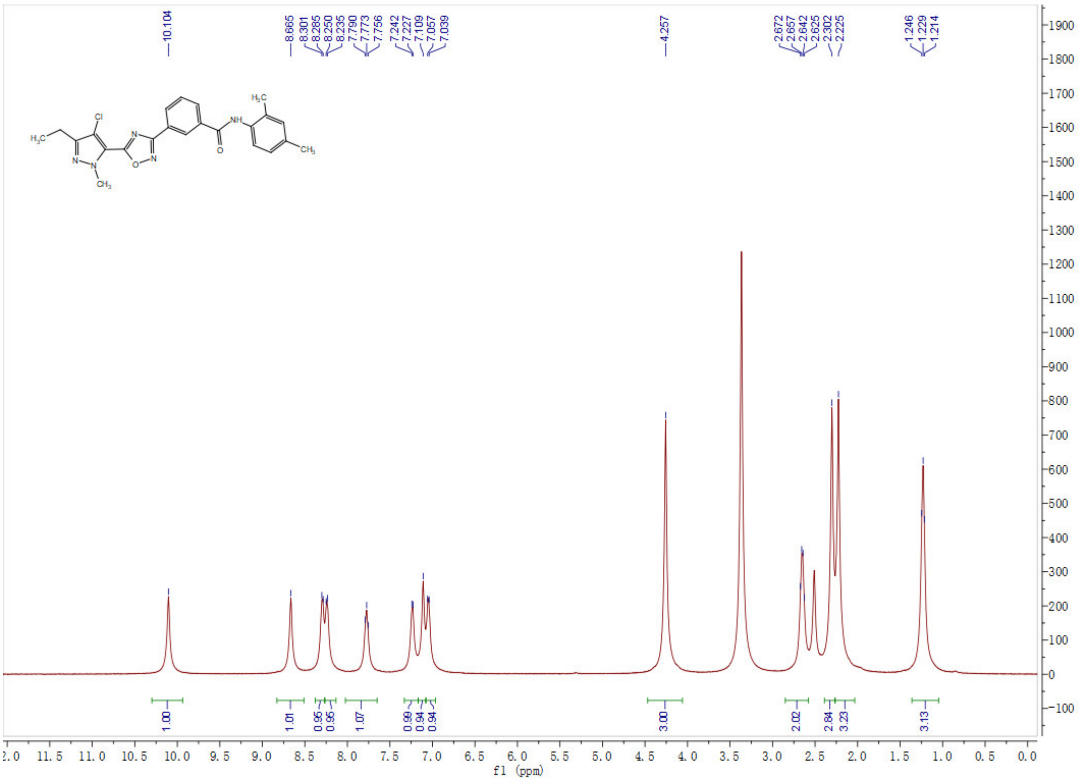

Figure S15. ^{1}H NMR spectra of **14o**

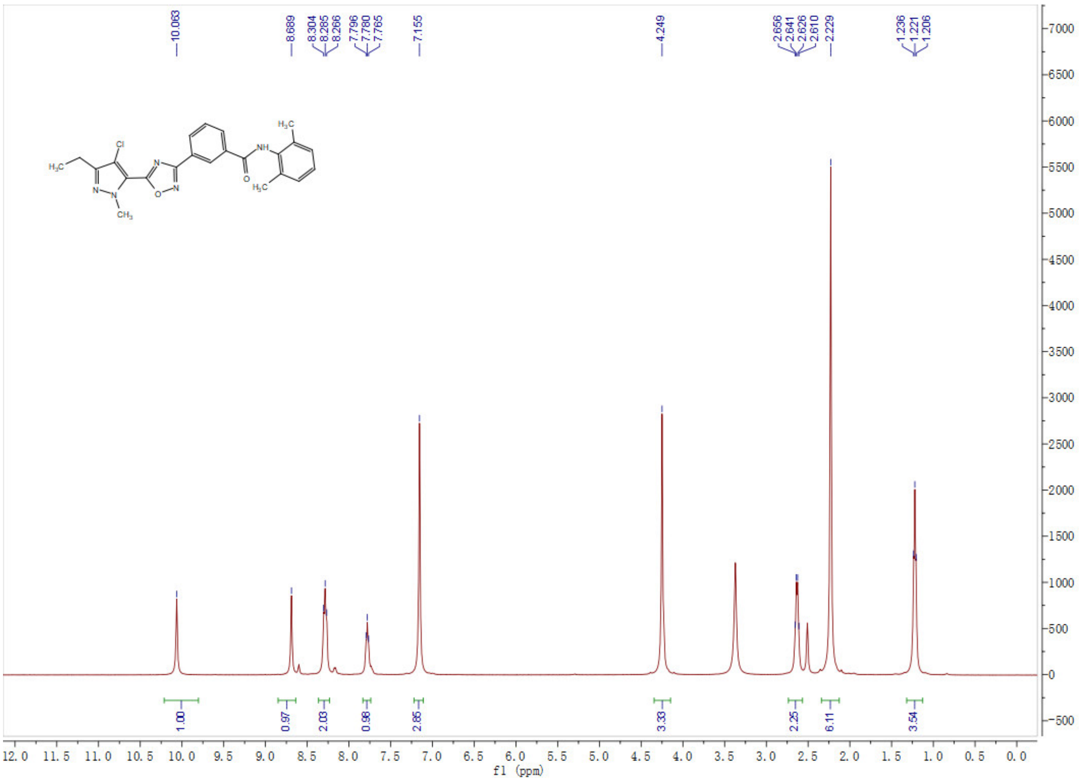

Figure S16. ^{1}H NMR spectra of **14p**

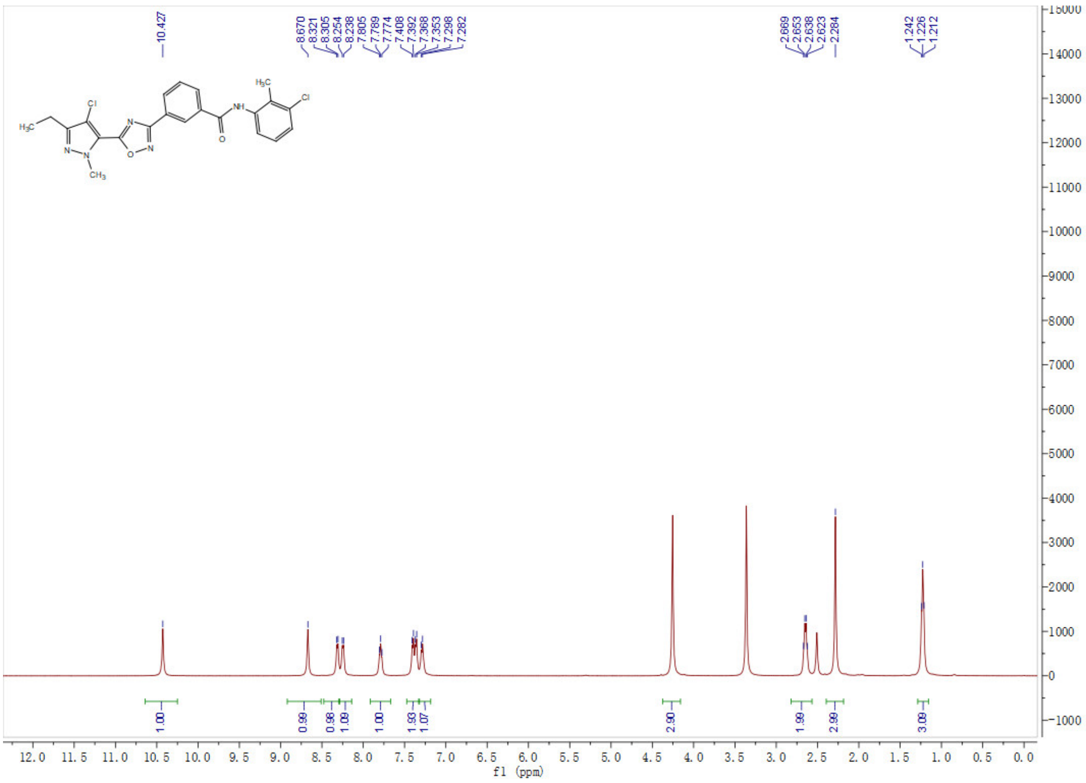

Figure S17. ^{1}H NMR spectra of **14q**

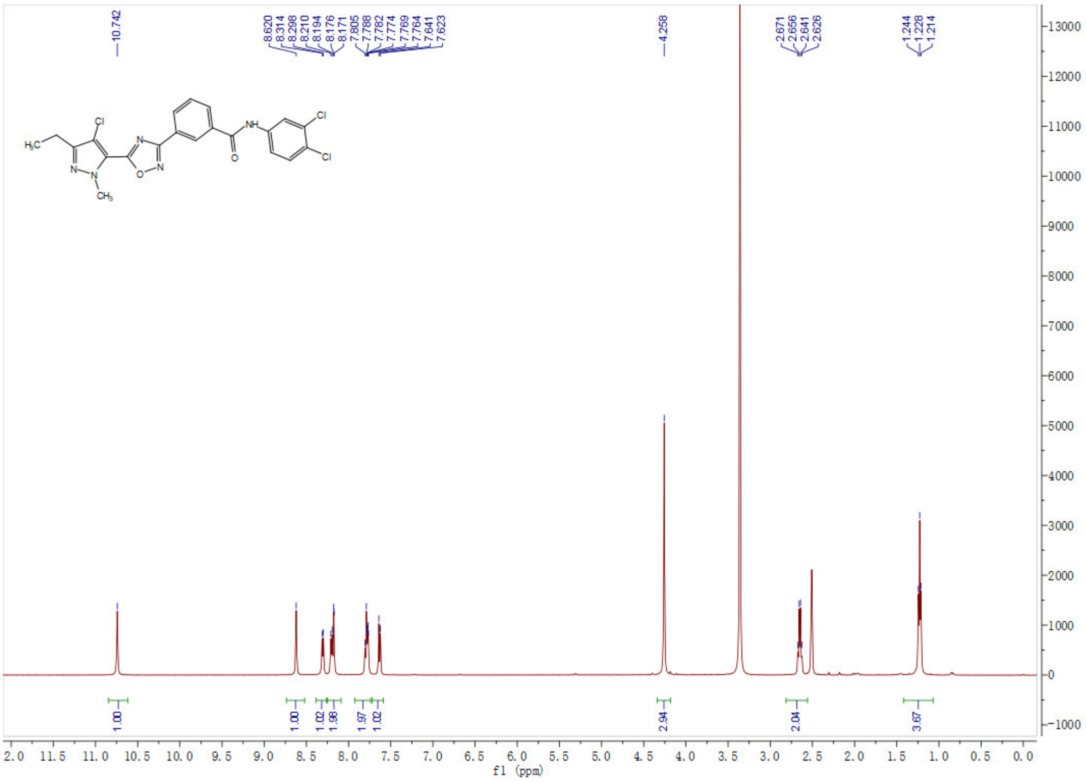

Figure S18. ^{1}H NMR spectra of **14r**

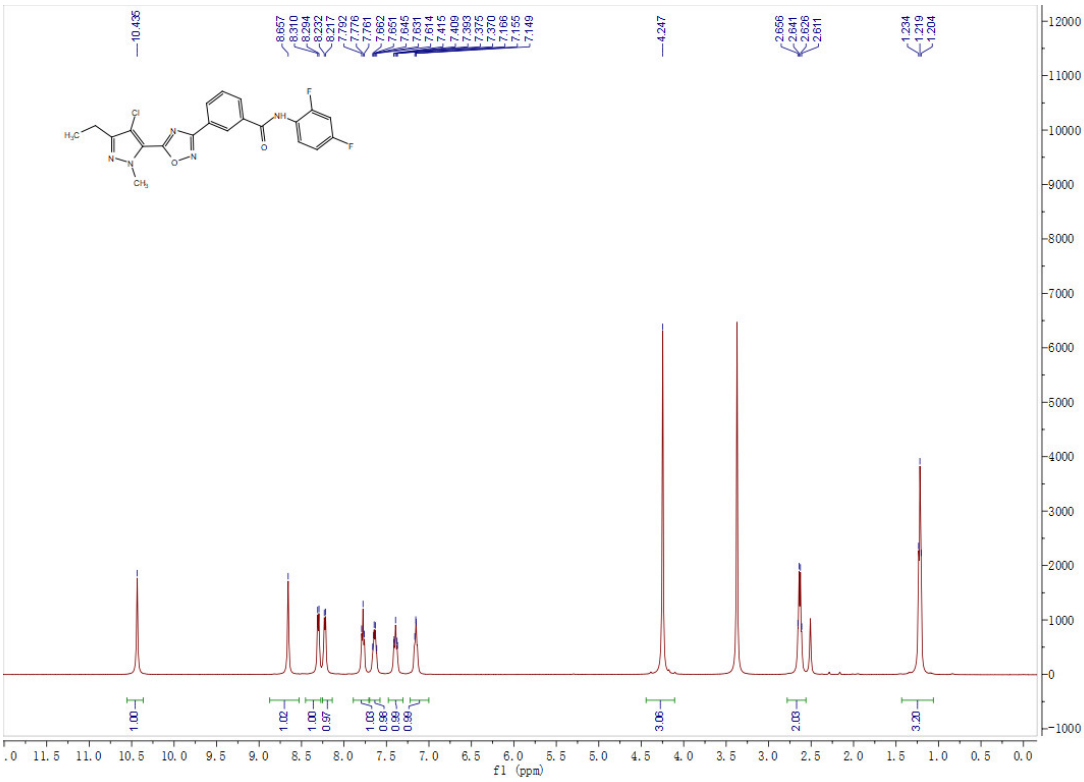

Figure S19. ^{1}H NMR spectra of **14s**

2. ^{13}C NMR spectra of **14a~14s**

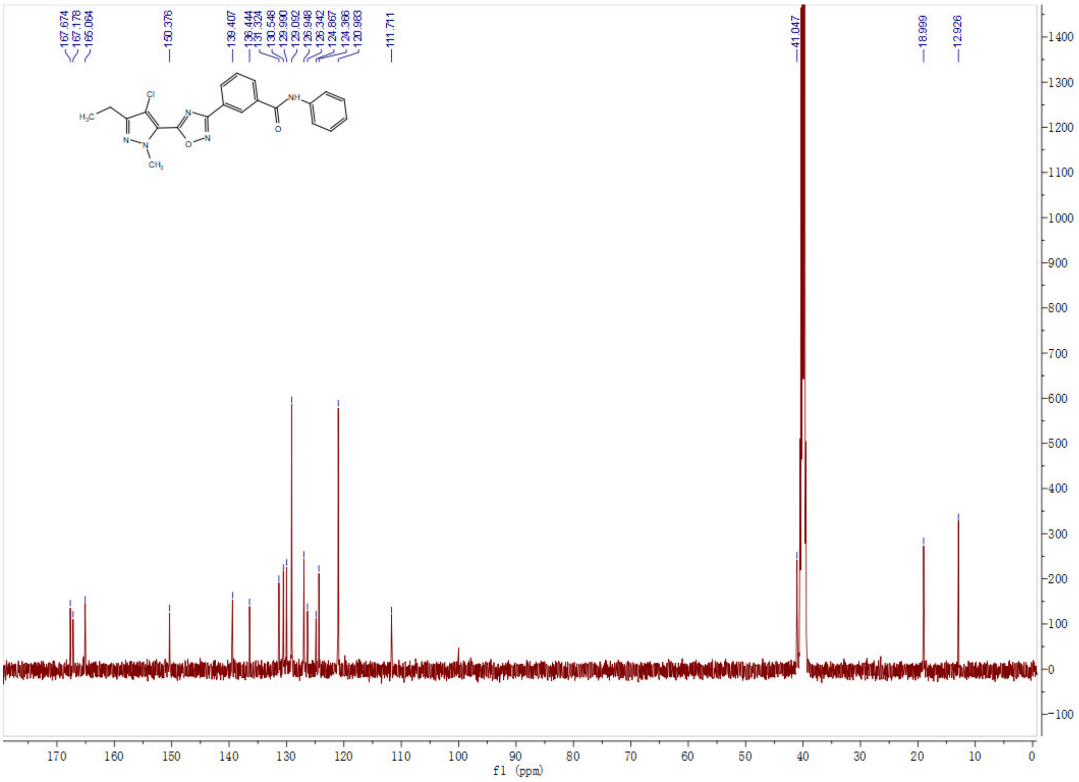

Figure S20. ^{13}C NMR spectra of **14a**

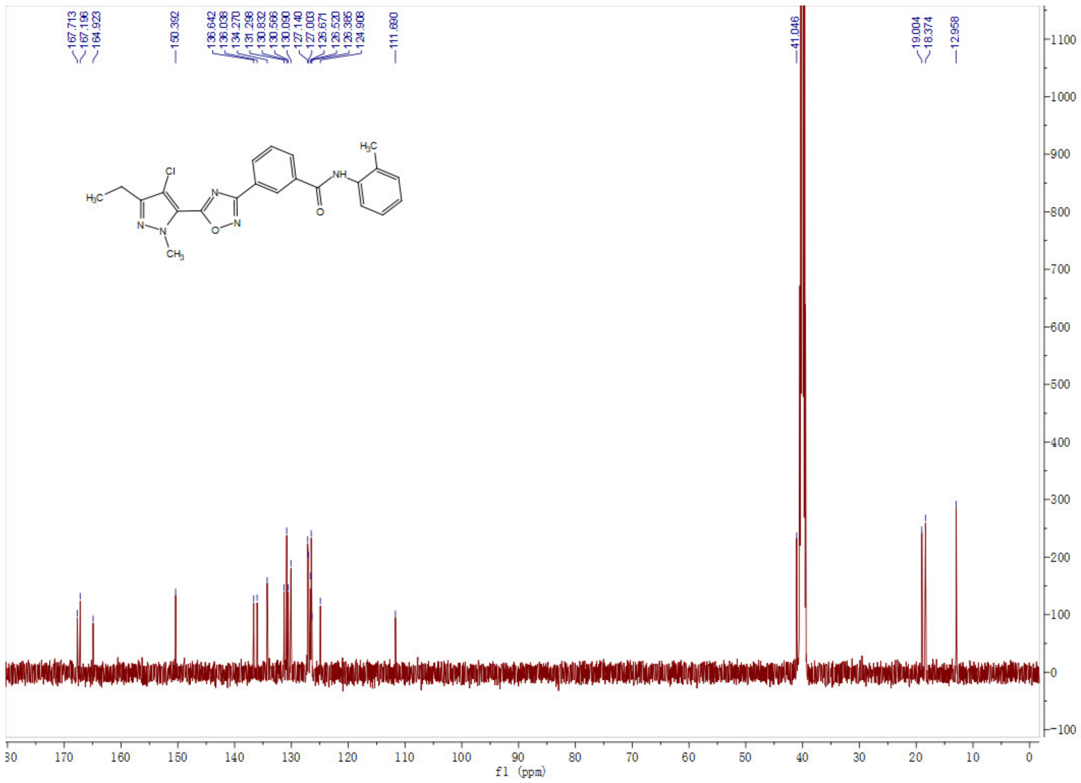

Figure S21. ^{13}C NMR spectra of **14b**

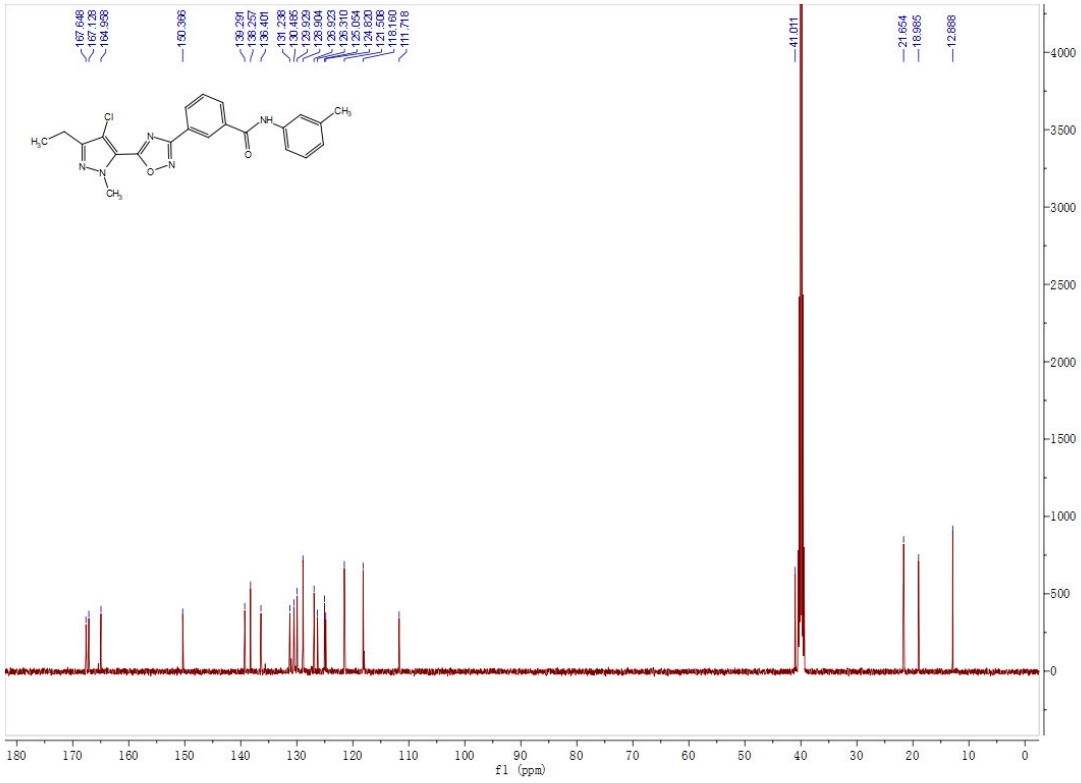

Figure S22. ^{13}C NMR spectra of **14c**

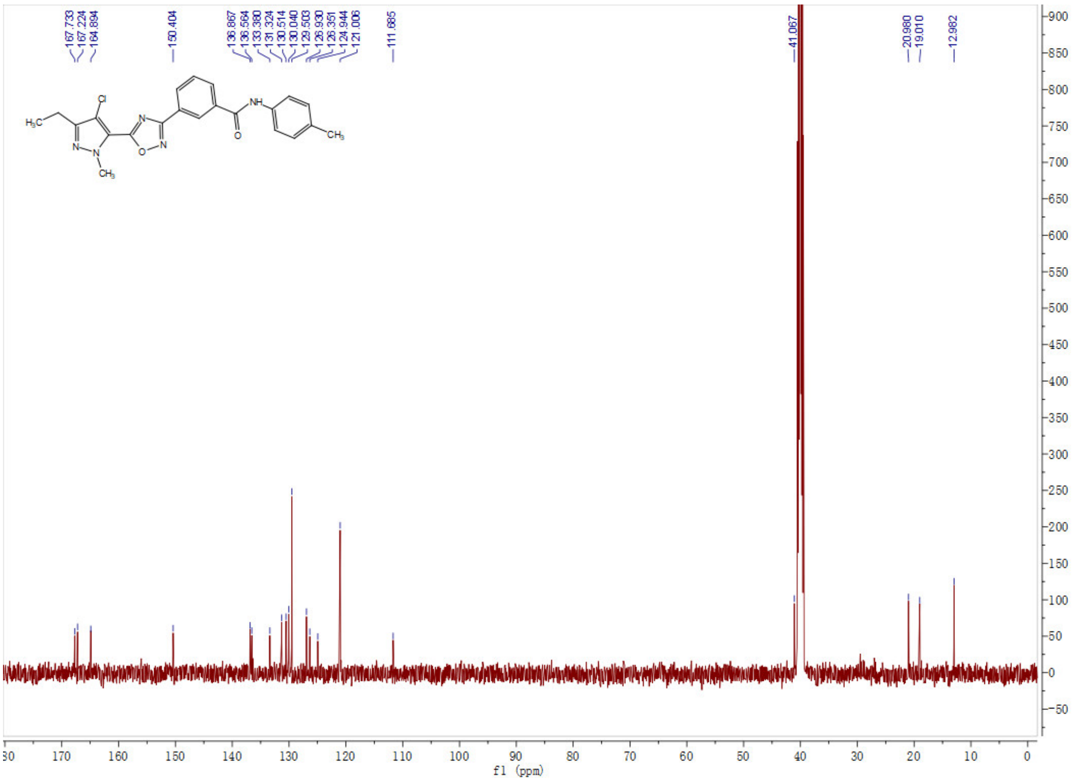

Figure S23. ^{13}C NMR spectra of **14d**

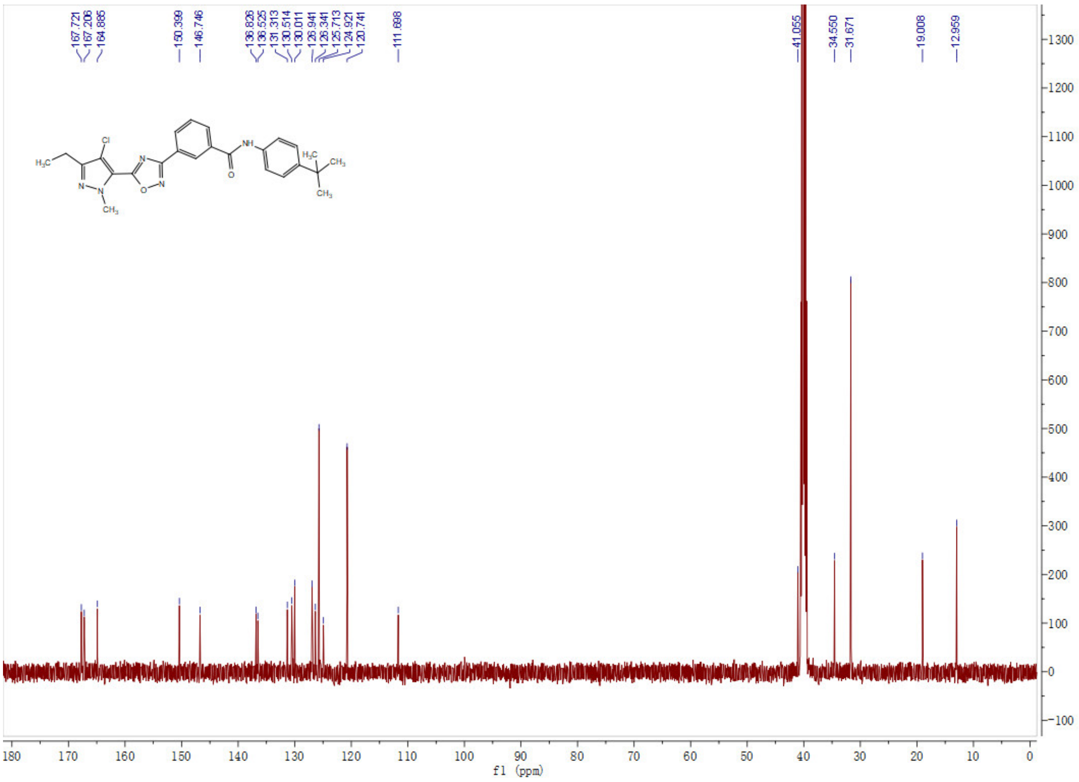

Figure S24. ^{13}C NMR spectra of **14e**

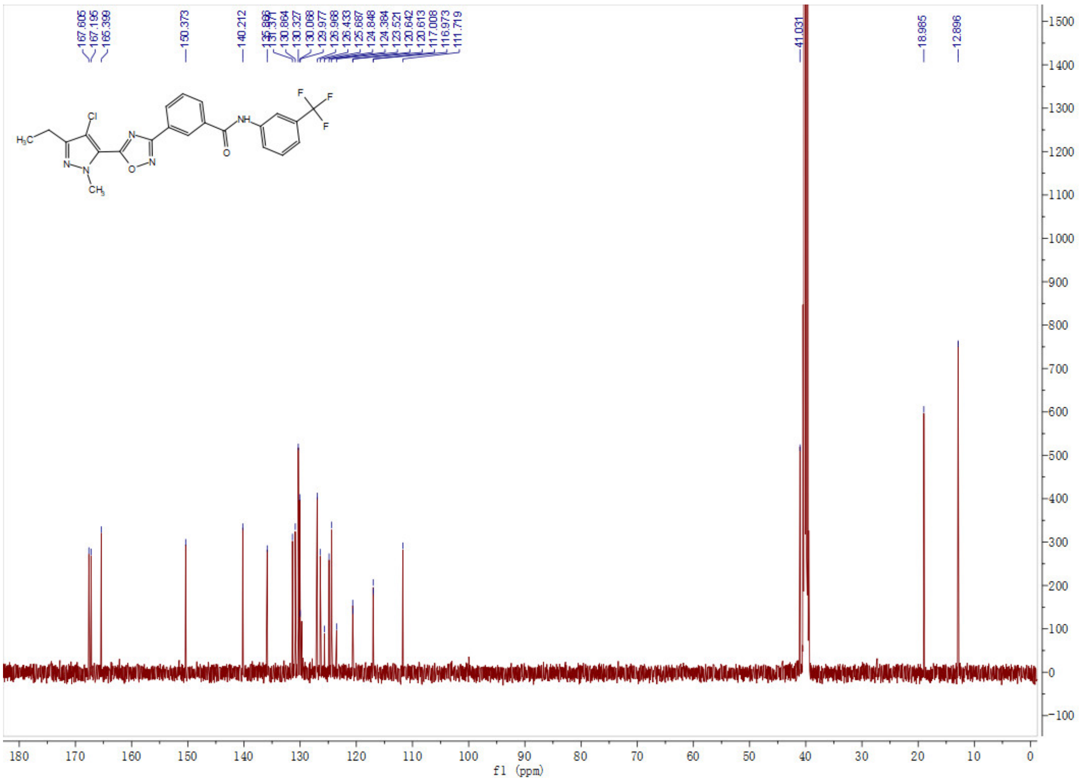

Figure S25. ^{13}C NMR spectra of **14f**

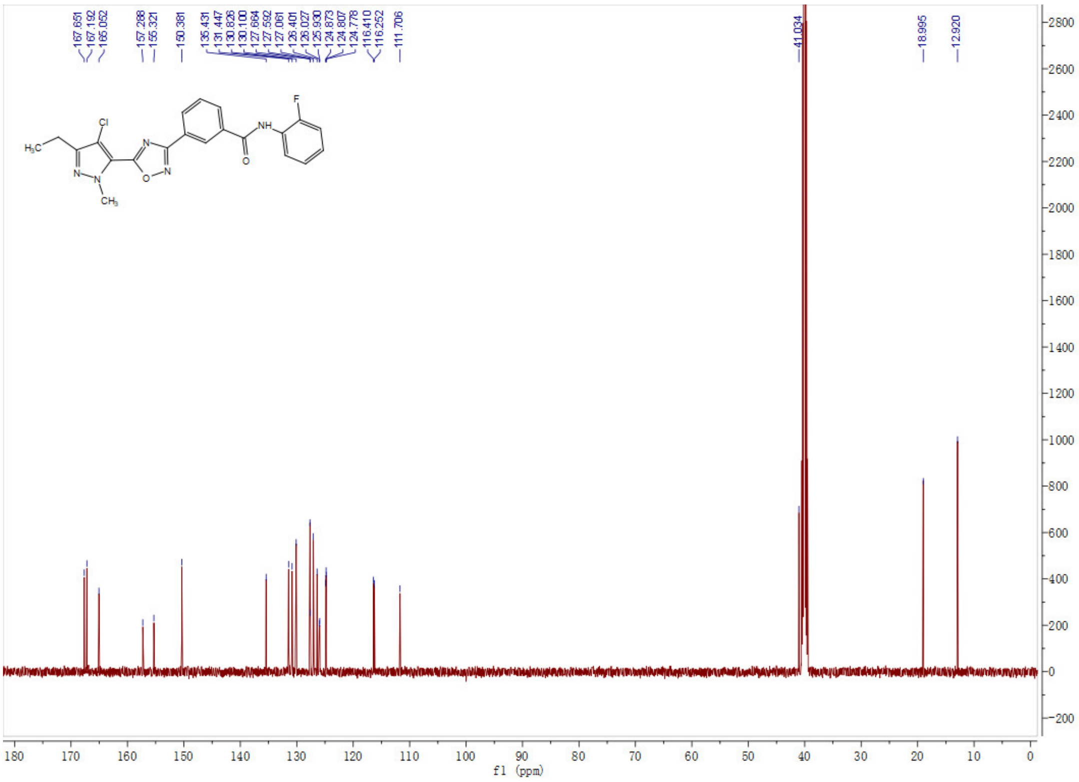

Figure S26. ^{13}C NMR spectra of **14g**

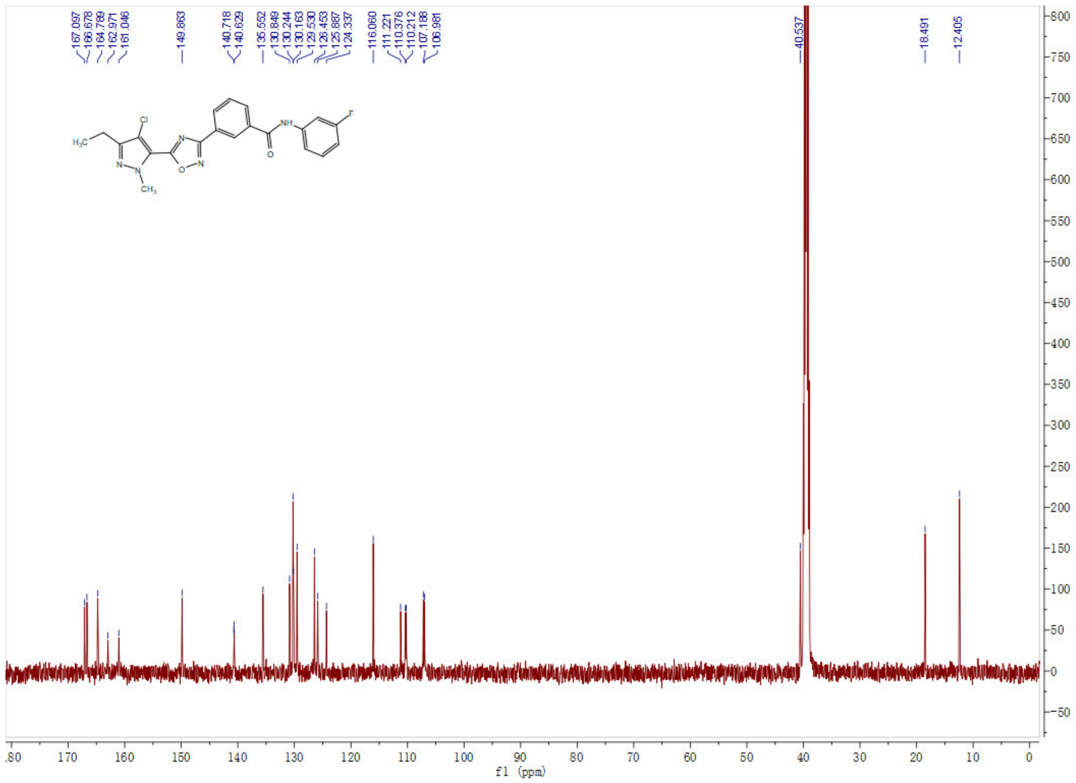

Figure S27. ^{13}C NMR spectra of **14h**

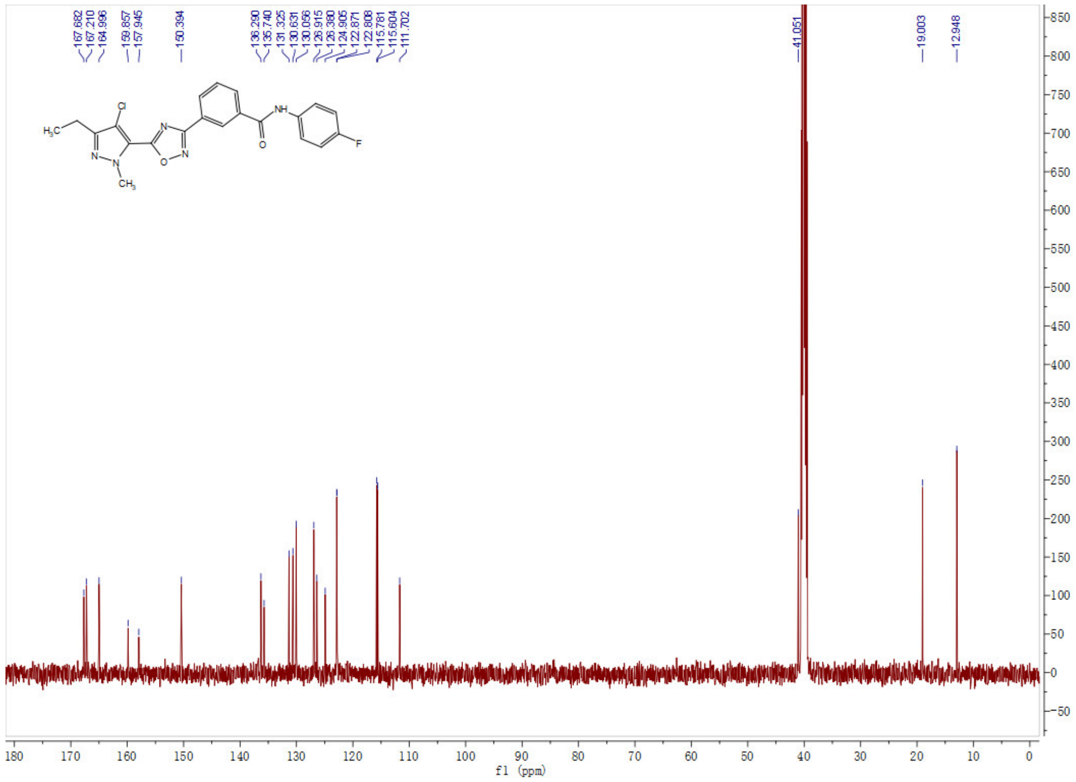

Figure S28. ^{13}C NMR spectra of **14i**

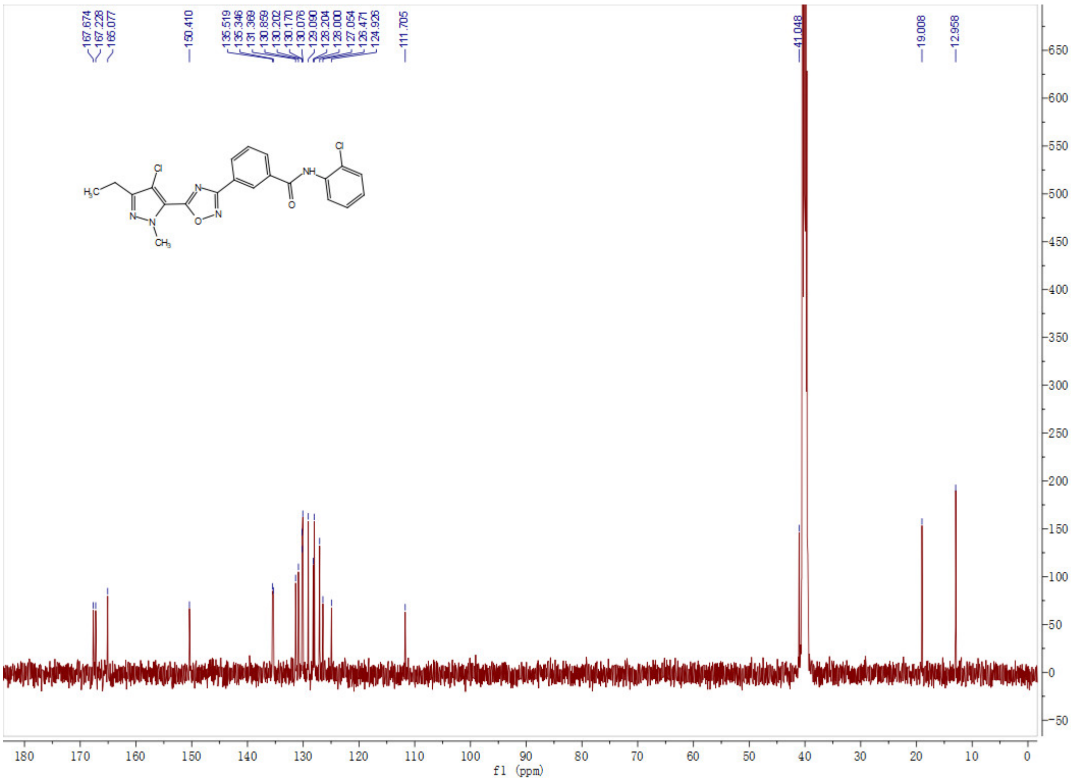

Figure S29. ^{13}C NMR spectra of **14j**

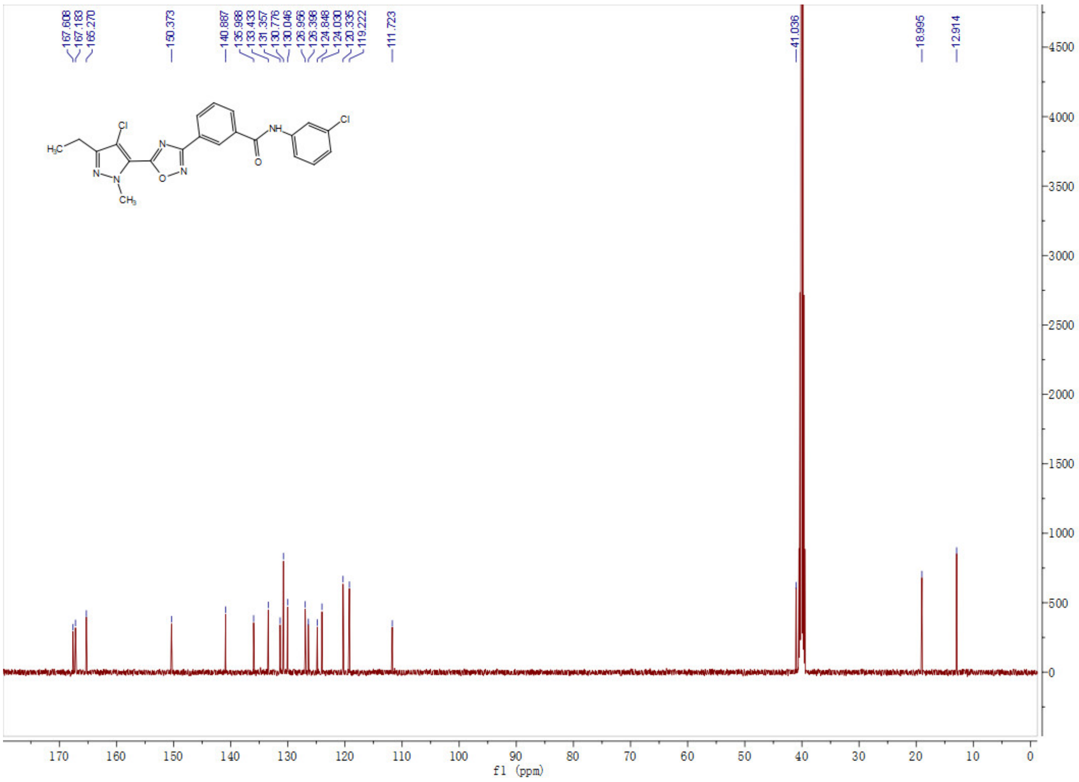

Figure S30. ^{13}C NMR spectra of **14k**

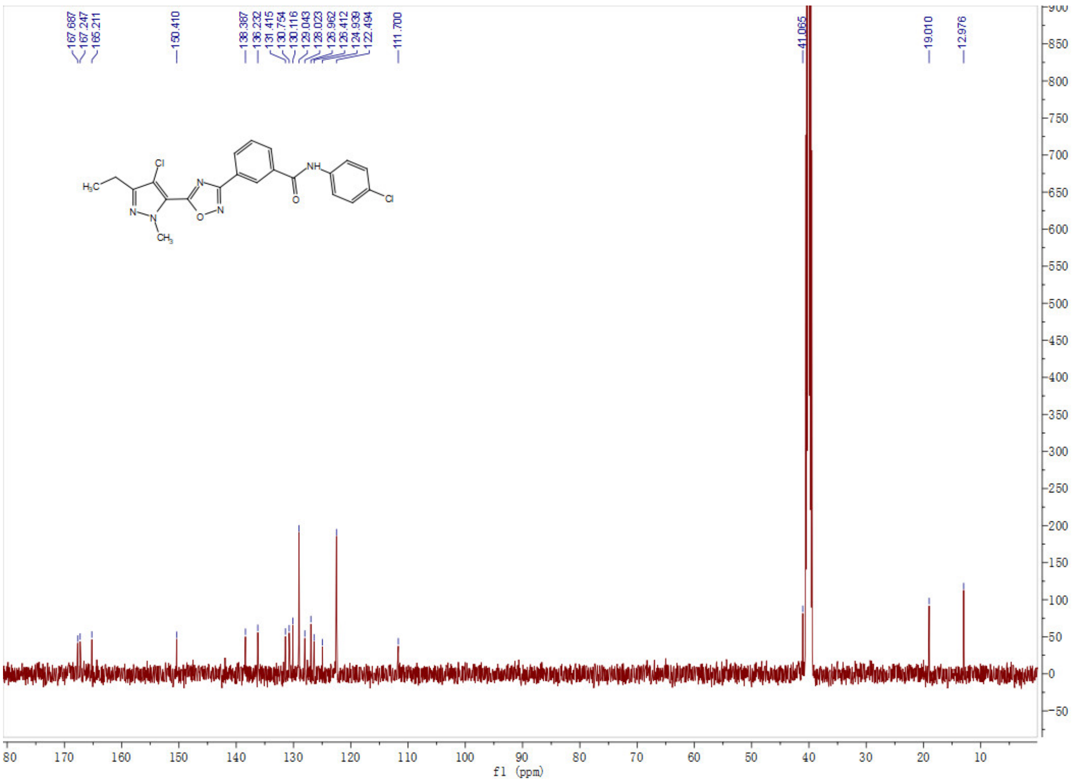

Figure S31. ^{13}C NMR spectra of **14l**

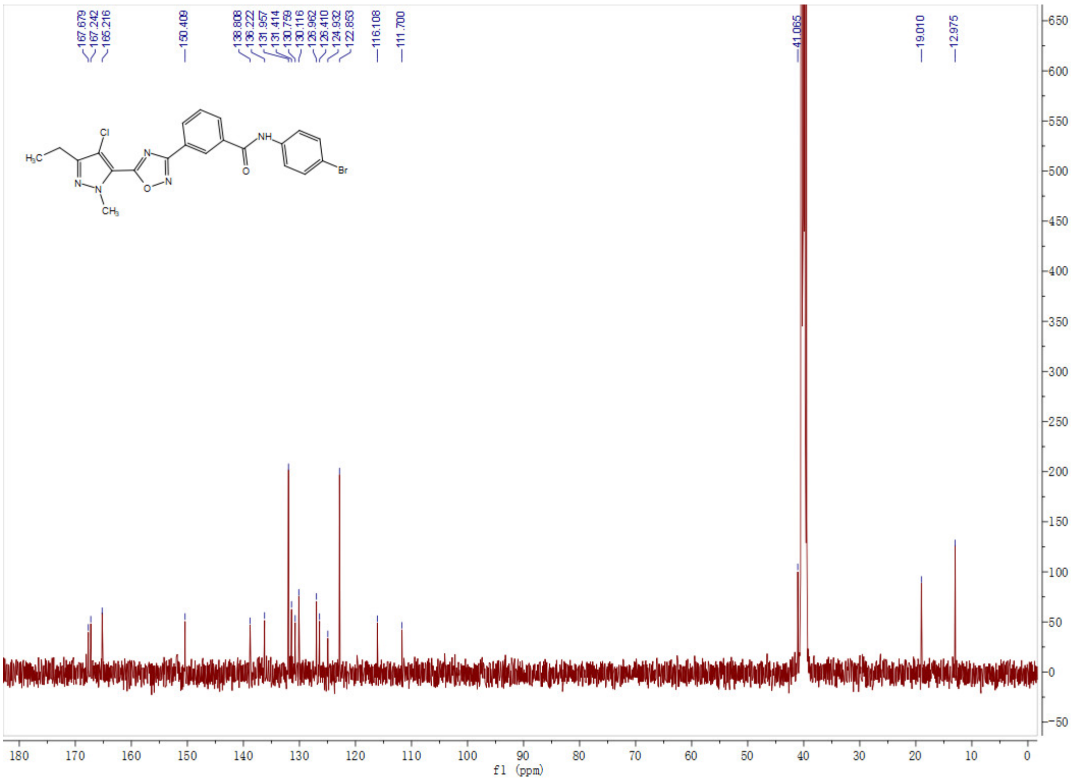

Figure S32. ^{13}C NMR spectra of **14m**

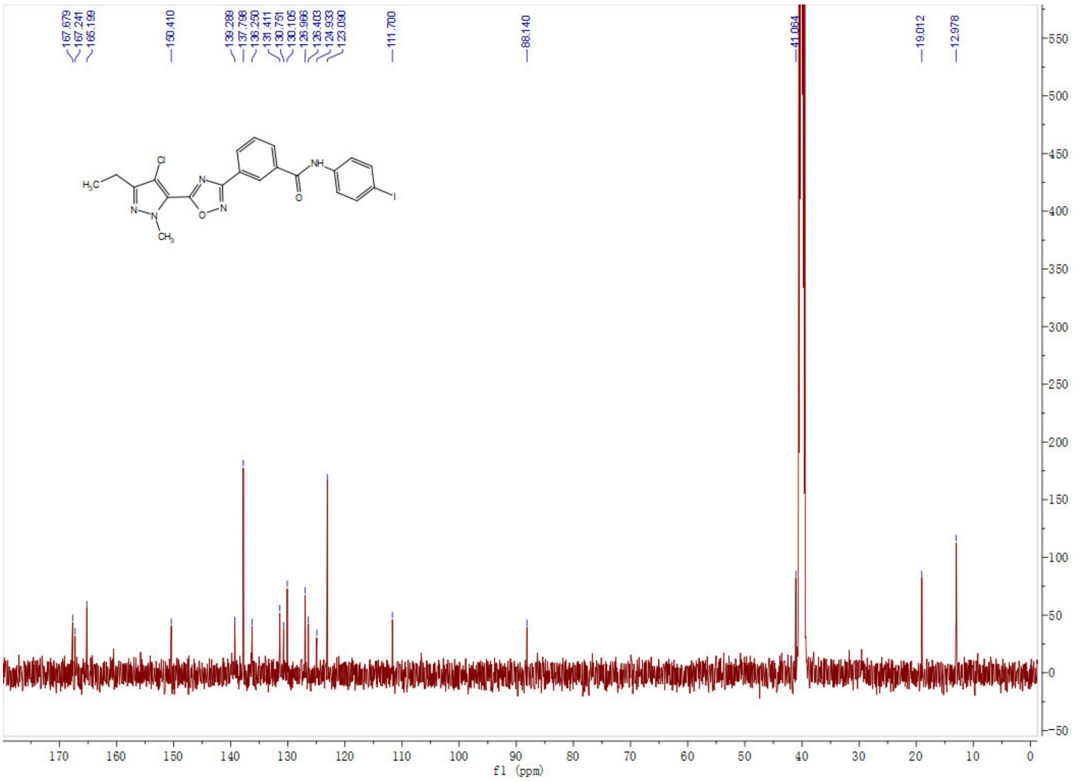

Figure S33. ^{13}C NMR spectra of **14n**

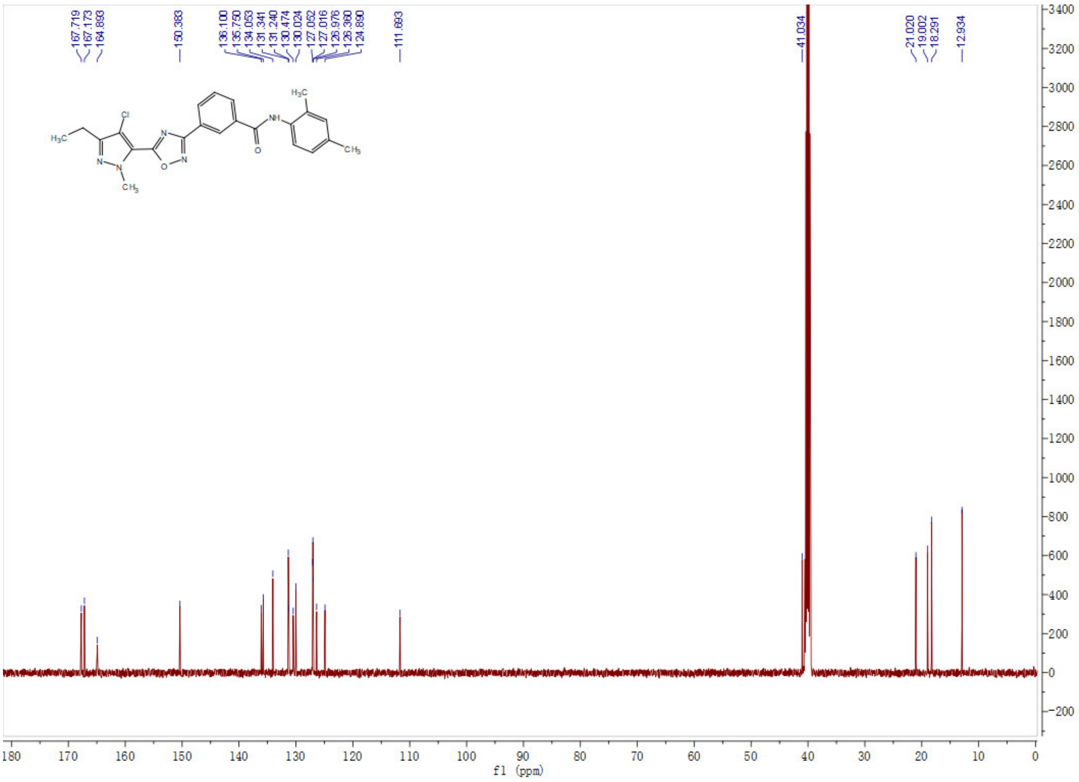

Figure S34. ^{13}C NMR spectra of **14o**

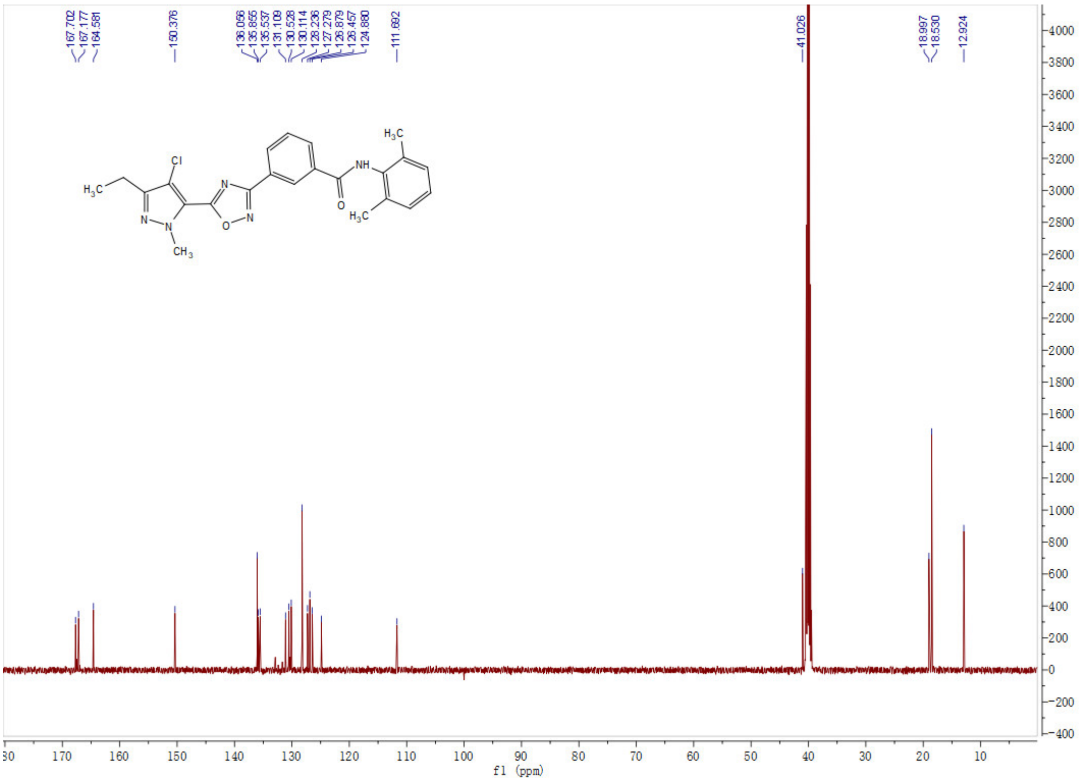

Figure S35. ^{13}C NMR spectra of **14p**

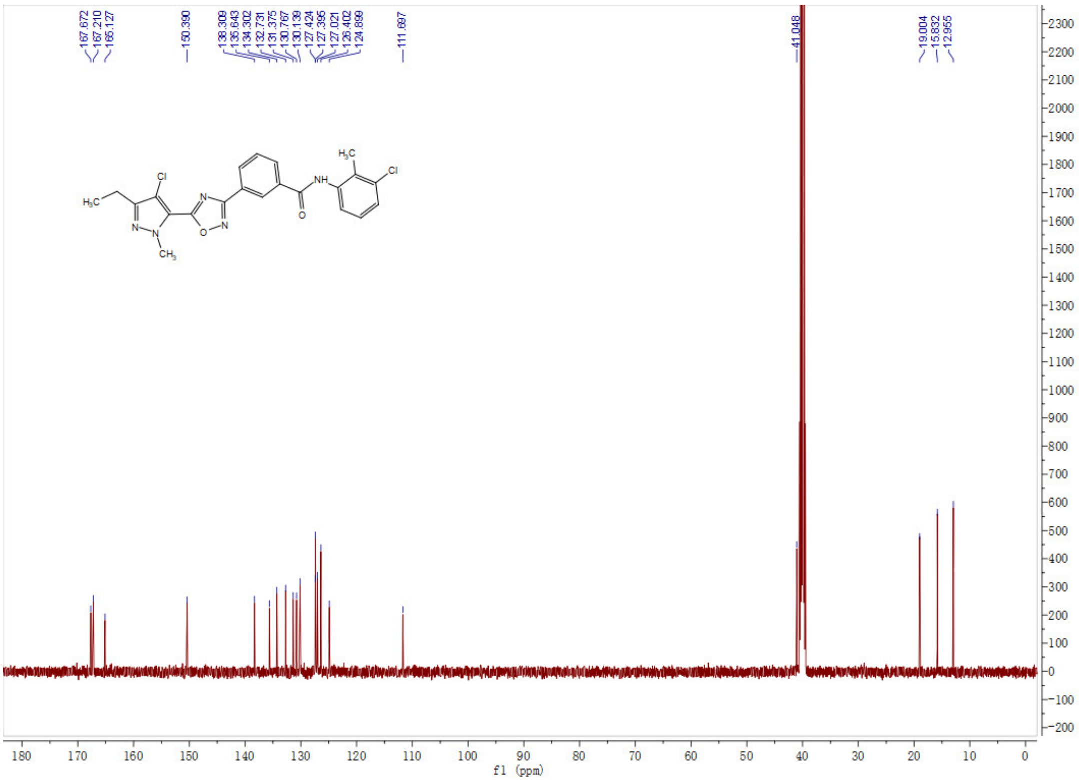

Figure S36. ^{13}C NMR spectra of **14q**

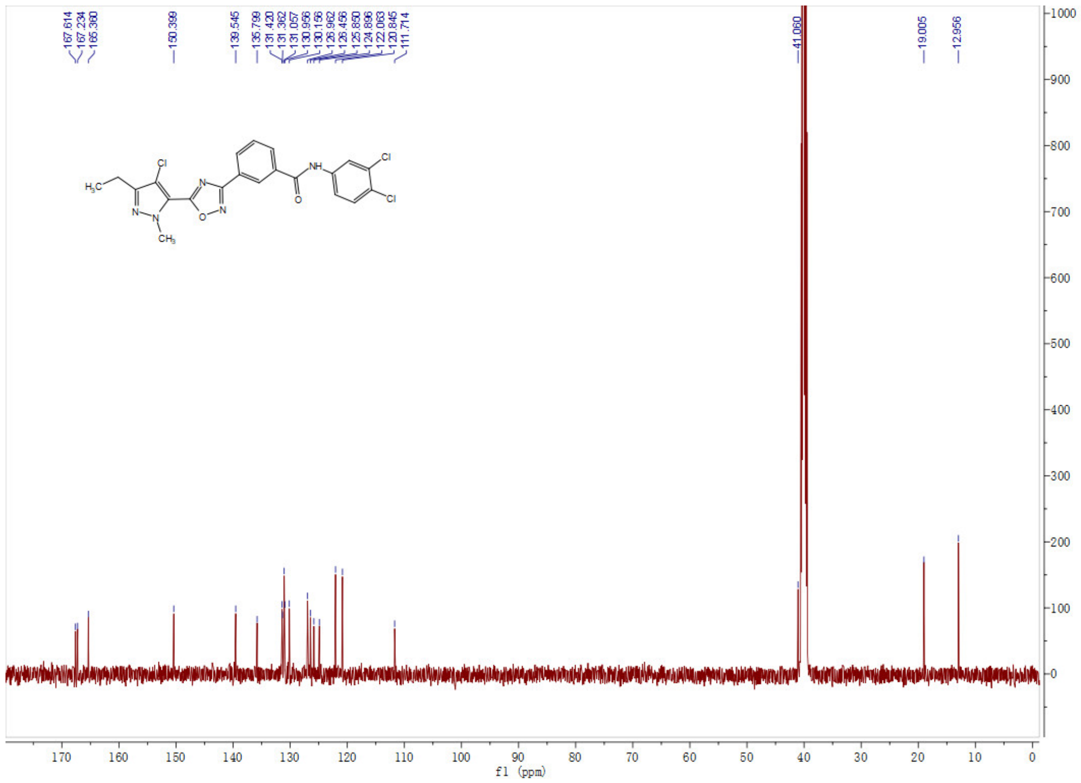

Figure S37. ^{13}C NMR spectra of **14r**

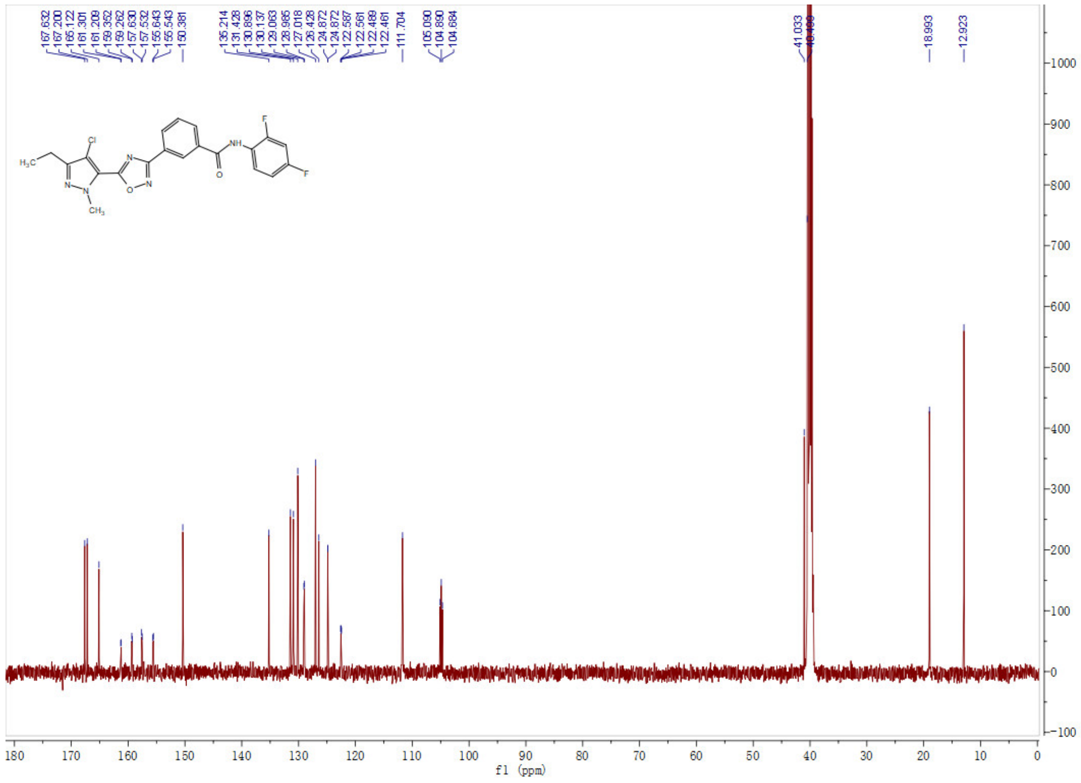

Figure S38. ^{13}C NMR spectra of **14s**

3. ESI-HRMS spectra of **14a~14s**

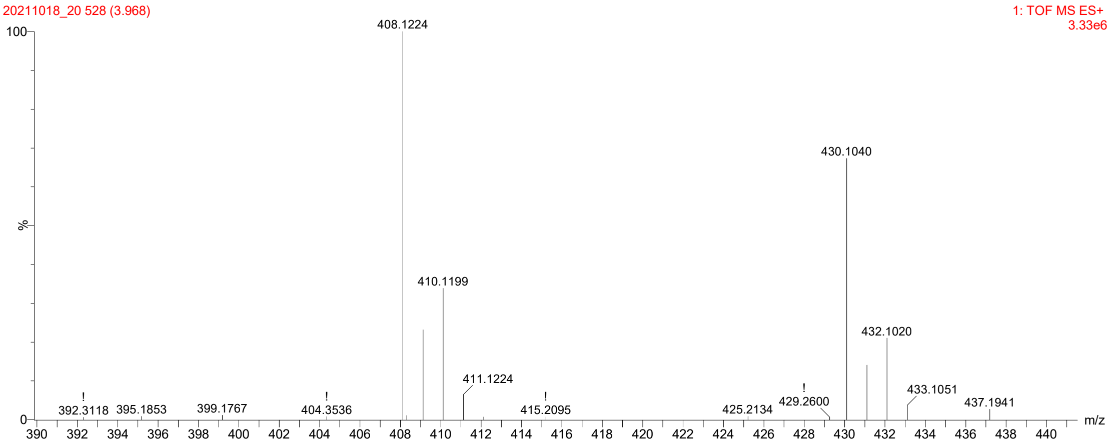

Figure S39. ESI-HRMS spectra of **14a**

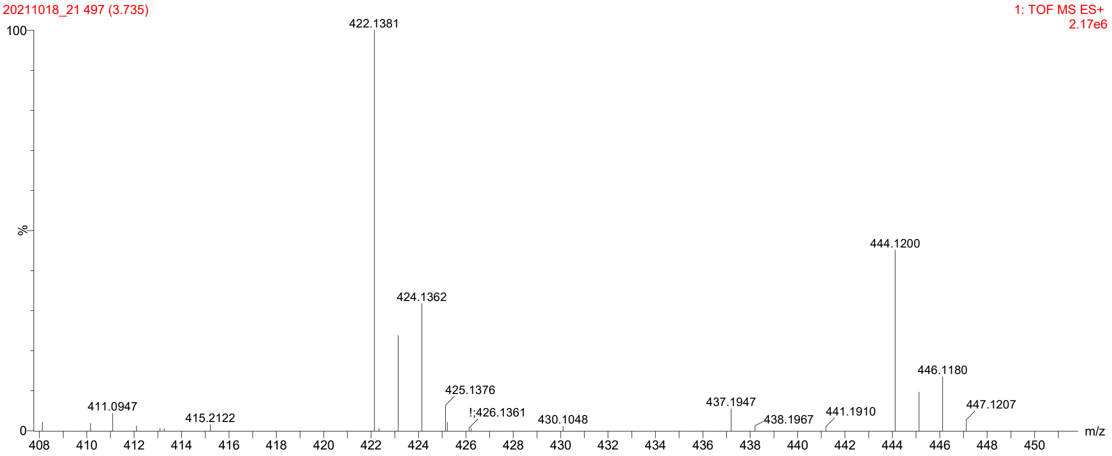

Figure S40. ESI-HRMS spectra of **14b**

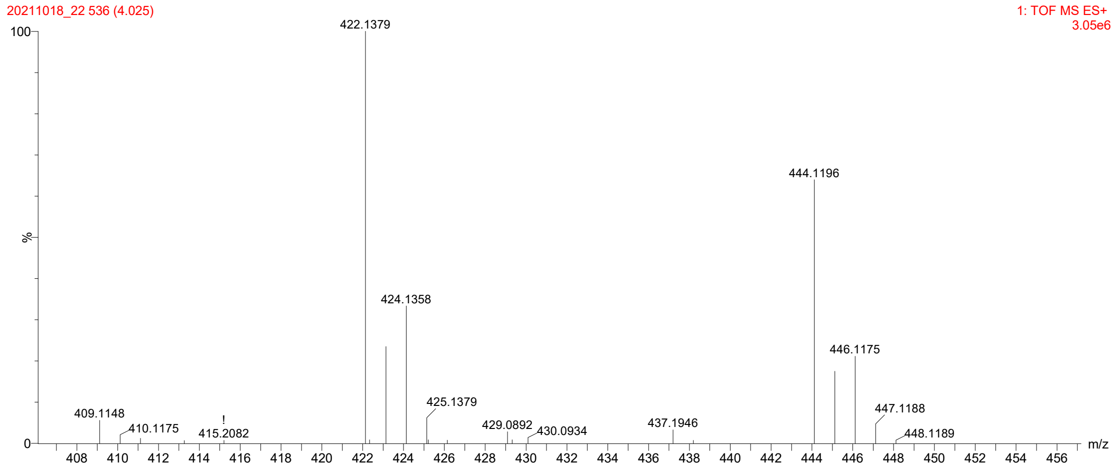

Figure S41. ESI-HRMS spectra of **14c**

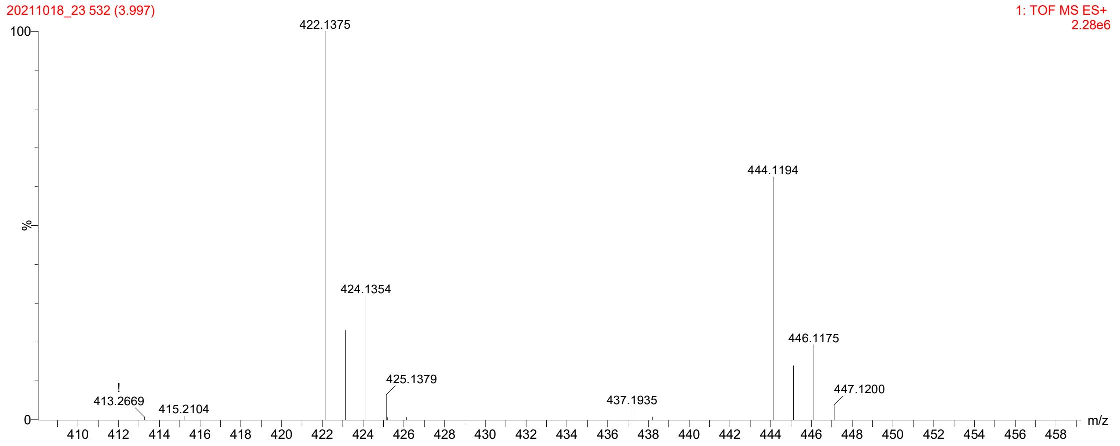

Figure S42. ESI-HRMS spectra of **14d**

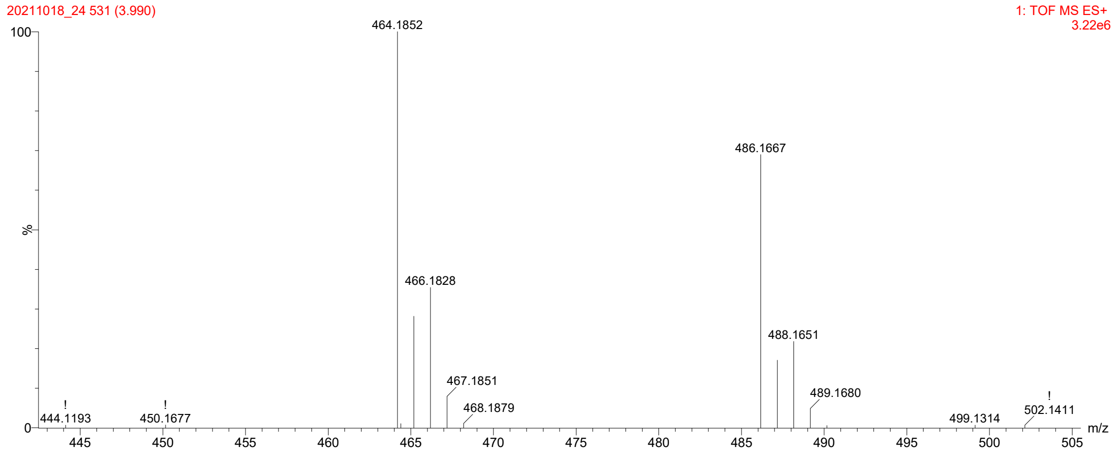

Figure S43. ESI-HRMS spectra of **14e**

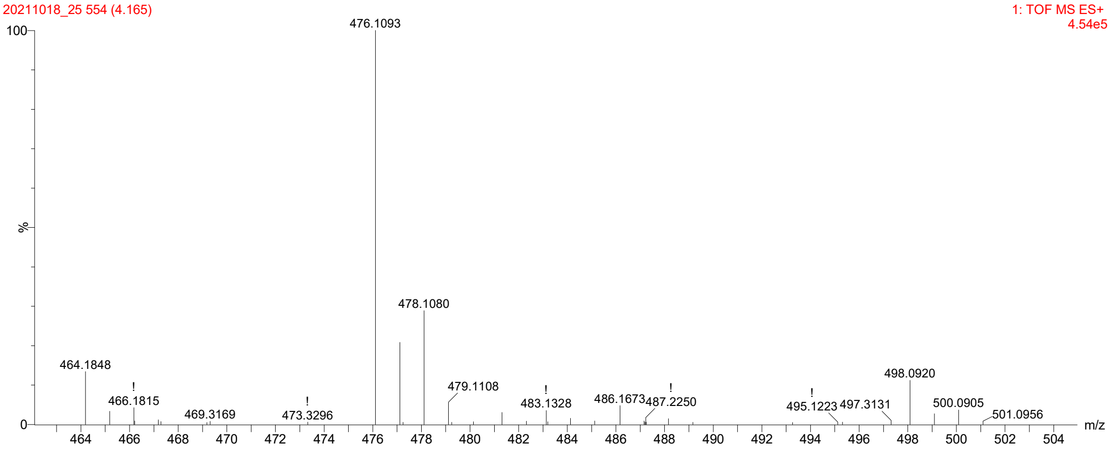

Figure S44. ESI-HRMS spectra of **14f**

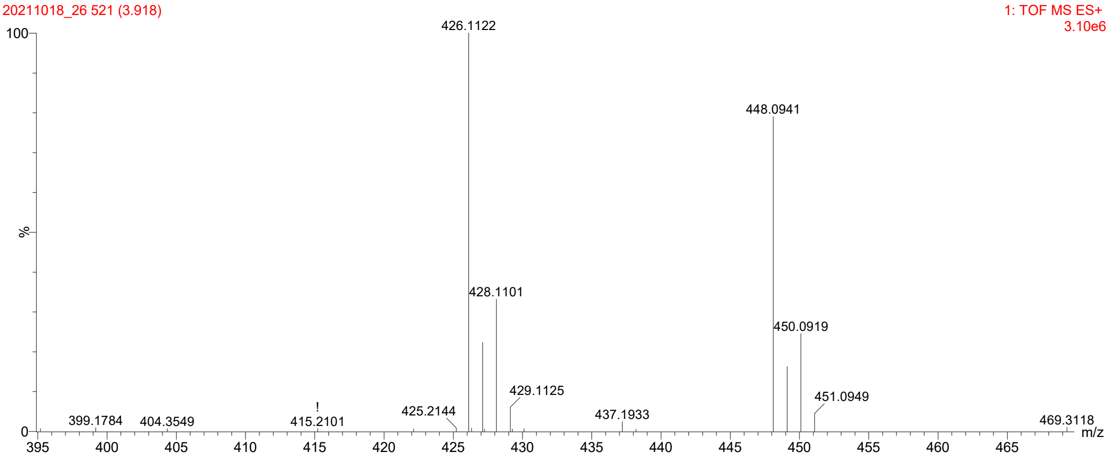

Figure S45. ESI-HRMS spectra of **14g**

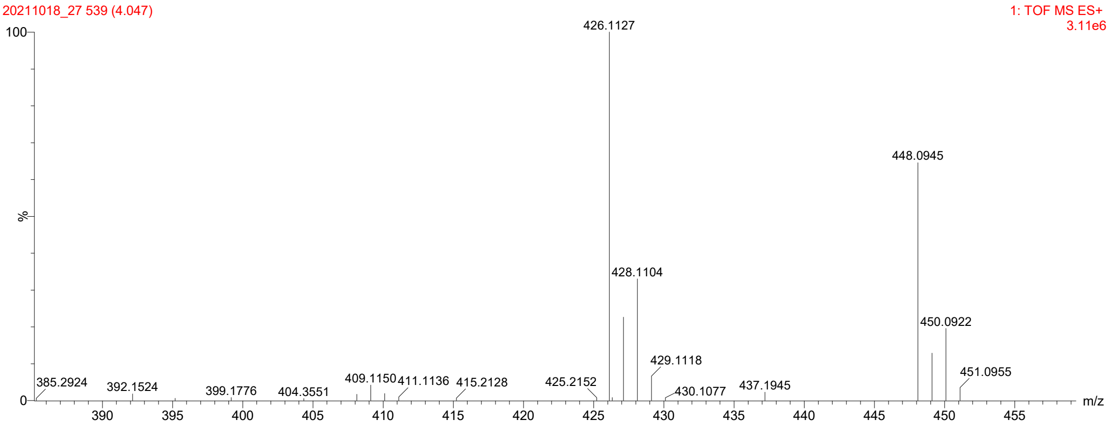

Figure S46. ESI-HRMS spectra of **14h**

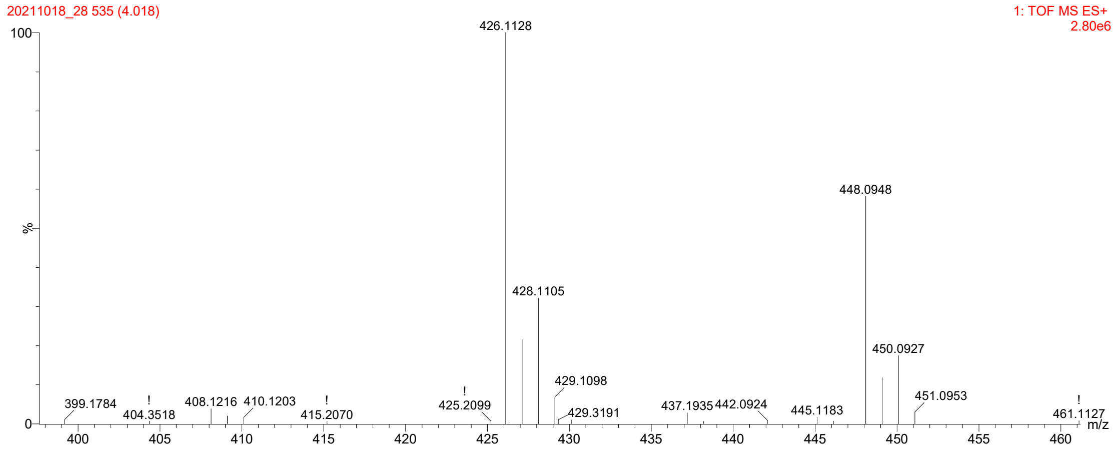

Figure S47. ESI-HRMS spectra of **14i**

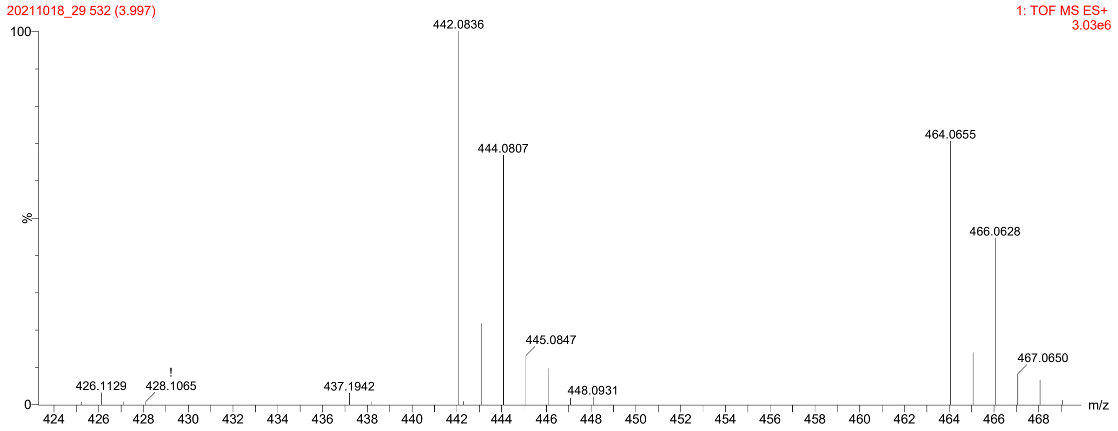

Figure S48. ESI-HRMS spectra of **14j**

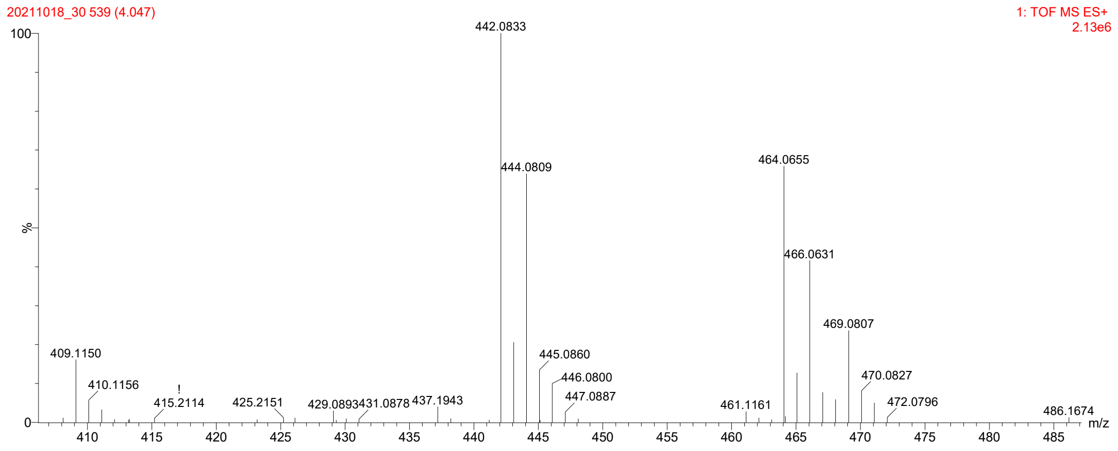

Figure S49. ESI-HRMS spectra of **14k**

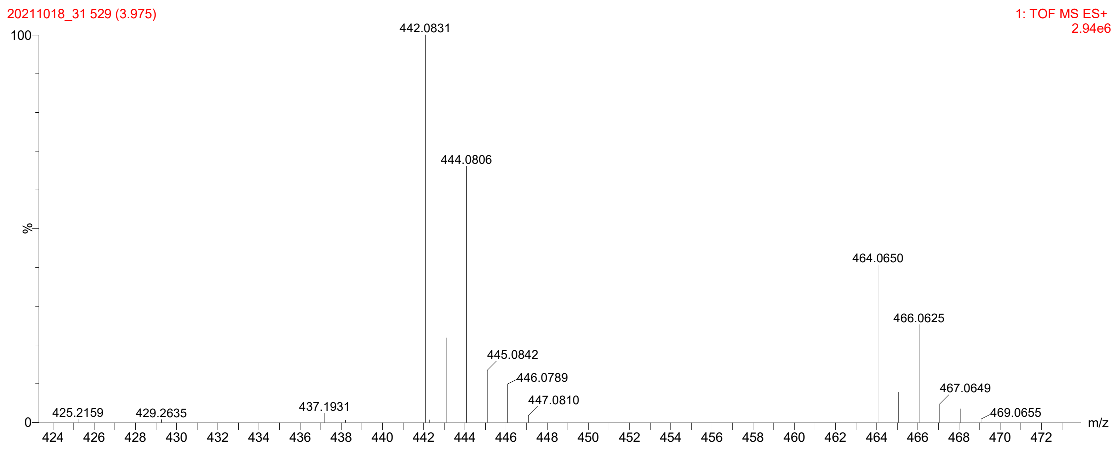

Figure S50. ESI-HRMS spectra of **14l**

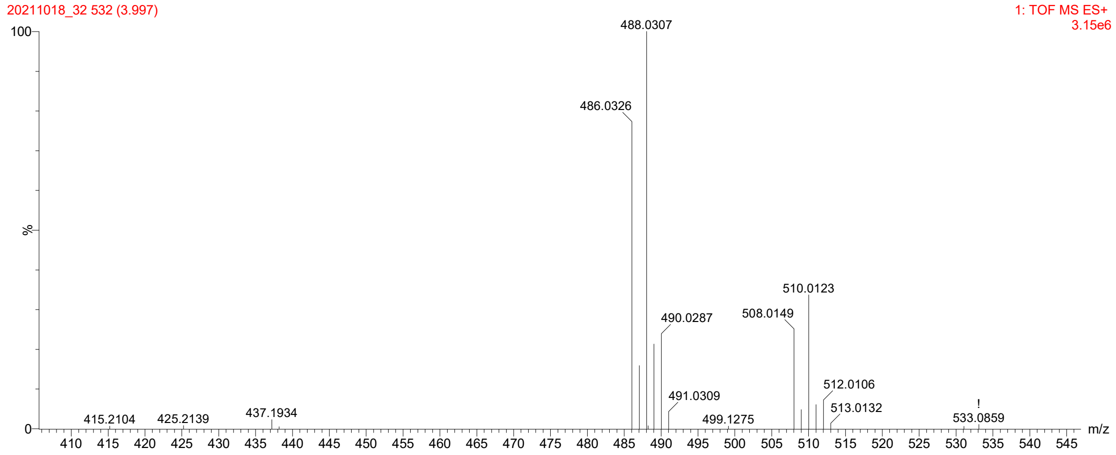

Figure S51. ESI-HRMS spectra of **14m**

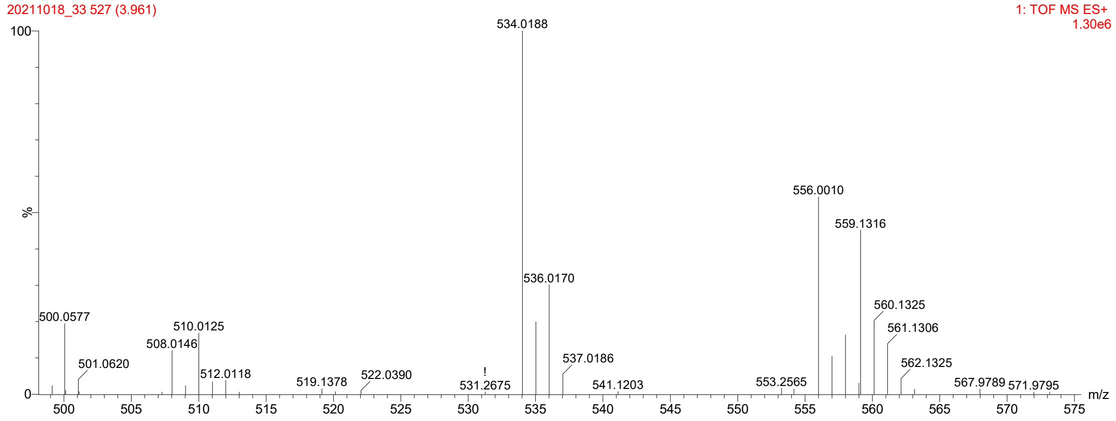

Figure S52. ESI-HRMS spectra of **14n**

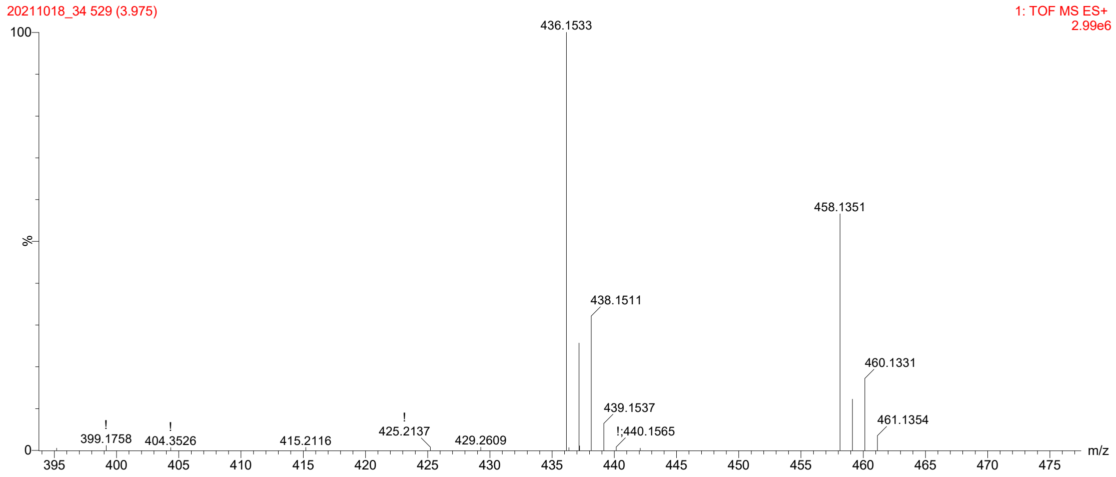

Figure S53. ESI-HRMS spectra of **14o**

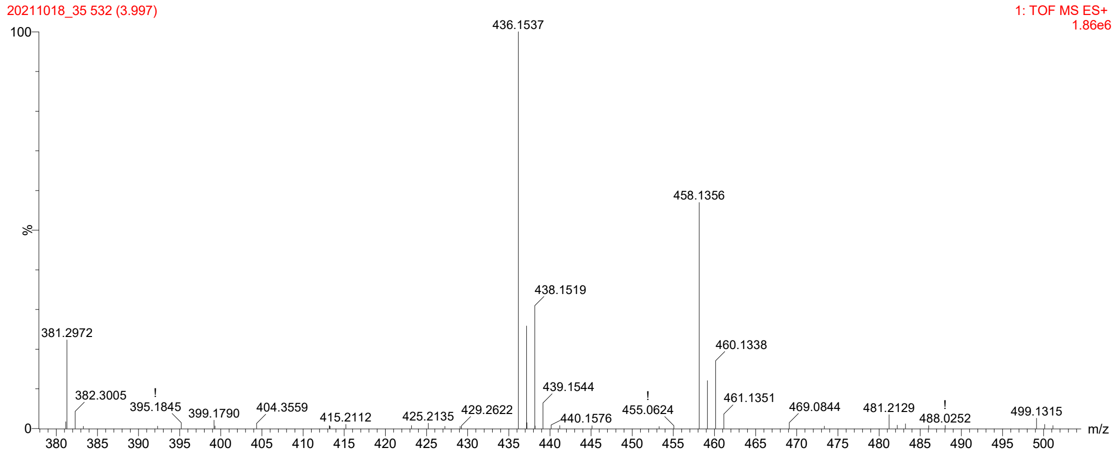

Figure S54. ESI-HRMS spectra of **14p**

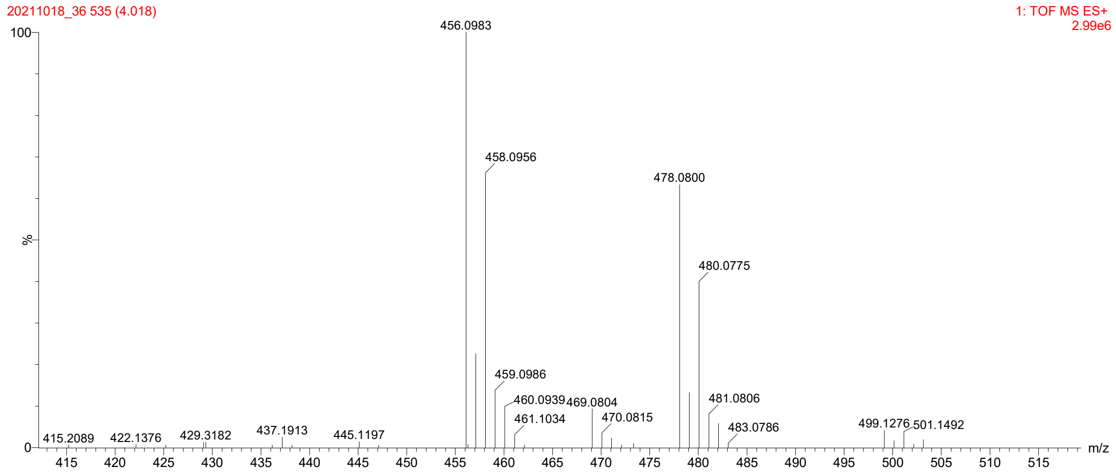

Figure S55. ESI-HRMS spectra of **14q**

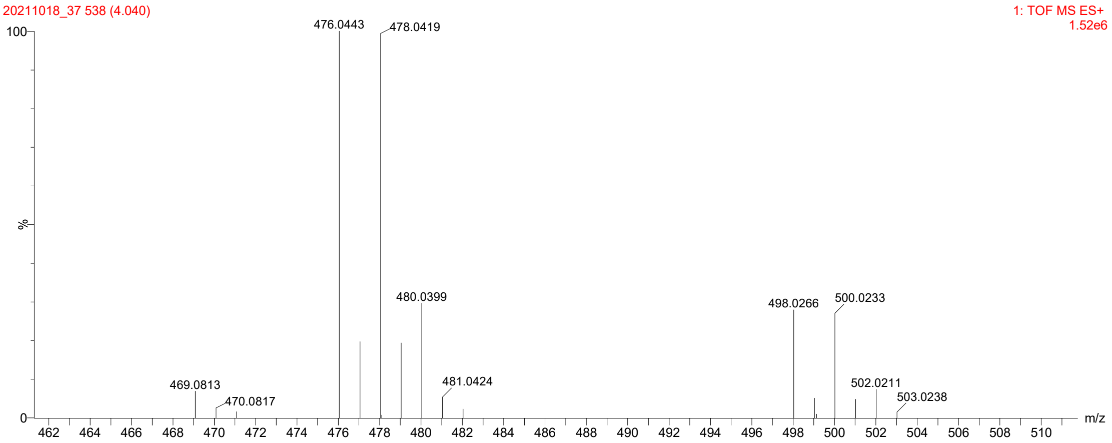

Figure S56. ESI-HRMS spectra of **14r**

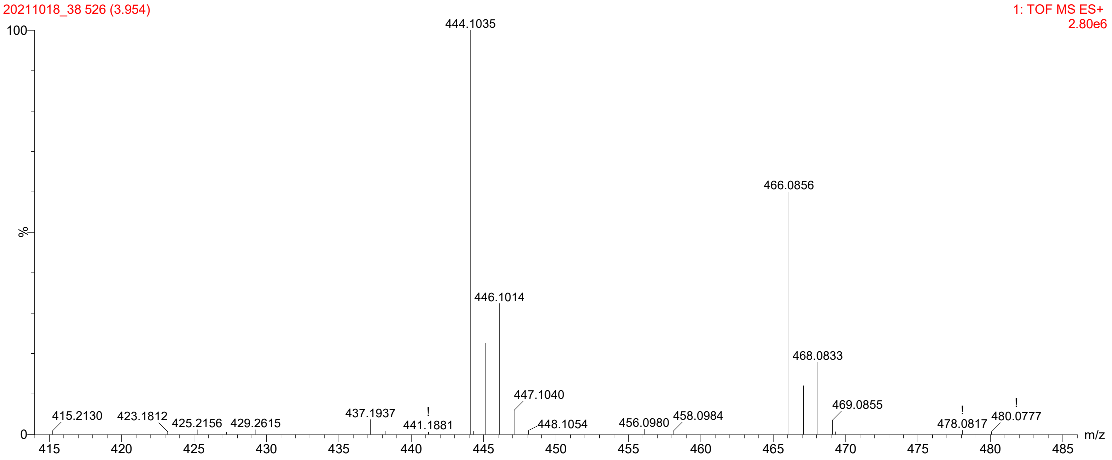

Figure S57. ESI-HRMS spectra of **14s**
